# Myeloid-specific HNRNPA2B1 deficiency disrupts macrophage function and in vivo responses

**DOI:** 10.1093/jimmun/vkaf073

**Published:** 2025-05-25

**Authors:** Mays Mohammed Salih, Chi G Weindel, Eric Malekos, Lisa Sudek, Sol Katzman, Cory J Mabry, Morgan J Chapman, Aja K Coleman, Sikandar Azam, Robert O Watson, Kristin L Patrick, Susan Carpenter

**Affiliations:** Department of Molecular, Cell and Developmental Biology, University of California Santa Cruz, Santa Cruz, CA, United States; Department of Microbial Pathogenesis and Immunology, College of Medicine, Texas A&M University, Bryan, TX, United States; Department of Molecular, Cell and Developmental Biology, University of California Santa Cruz, Santa Cruz, CA, United States; Department of Molecular, Cell and Developmental Biology, University of California Santa Cruz, Santa Cruz, CA, United States; Department of Molecular, Cell and Developmental Biology, University of California Santa Cruz, Santa Cruz, CA, United States; Department of Microbial Pathogenesis and Immunology, College of Medicine, Texas A&M University, Bryan, TX, United States; Department of Pathology, Microbiology, and Immunology, Vanderbilt University School of Medicine, Nashville, TN, United States; Department of Microbial Pathogenesis and Immunology, College of Medicine, Texas A&M University, Bryan, TX, United States; Department of Pathology, Microbiology, and Immunology, Vanderbilt University School of Medicine, Nashville, TN, United States; Department of Microbial Pathogenesis and Immunology, College of Medicine, Texas A&M University, Bryan, TX, United States; Department of Pathology, Microbiology, and Immunology, Vanderbilt University School of Medicine, Nashville, TN, United States; Department of Microbial Pathogenesis and Immunology, College of Medicine, Texas A&M University, Bryan, TX, United States; Department of Microbial Pathogenesis and Immunology, College of Medicine, Texas A&M University, Bryan, TX, United States; Department of Pathology, Microbiology, and Immunology, Vanderbilt University School of Medicine, Nashville, TN, United States; Department of Microbial Pathogenesis and Immunology, College of Medicine, Texas A&M University, Bryan, TX, United States; Department of Pathology, Microbiology, and Immunology, Vanderbilt University School of Medicine, Nashville, TN, United States; Department of Molecular, Cell and Developmental Biology, University of California Santa Cruz, Santa Cruz, CA, United States

**Keywords:** gene regulation, inflammation, knockout mice, lipopolysaccharide, monocytes/macrophages

## Abstract

The mechanisms through which heterogeneous nuclear ribonucleoprotein A2B1 (HNRNPA2B1) contributes to innate immune gene regulation are poorly understood. To fill this gap, we generated a myeloid lineage–specific HNRNPA2B1-conditional mouse using LysMCre. In an endotoxic shock model, HNRNPA2B1-deficient mice exhibit dampened expression of inflammatory mediators despite increased infiltration of macrophages and neutrophils. Likewise, during infection with the gram-negative bacterial pathogen *Salmonella enterica*, HNRNPA2B1-deficient mice fail to mount protective inflammatory responses and experience higher bacterial burdens. To better understand the molecular mechanisms driving these phenotypes in vivo, we performed transcriptomics analysis of LPS-treated HNRNPA2B1-deficient macrophages ex vivo. We noted an increase in transcripts encoding nonproductive isoforms of a number of Interferon (IFN)-regulated genes, including the IFNG receptor (IFNGR). Focusing on IFNGR, we confirmed lower surface expression on HNRNPA2B1-deficient macrophages and dampened responsiveness in response to IFNG treatment. In conclusion, our data demonstrates that HNRNPA2B1 is essential for optimal macrophage function, particularly in the context of intracellular bacterial restriction in the case of *Salmonella* infection. This highlights a previously unappreciated role for RNA-binding proteins in mounting effective immune defenses.

## Teaser

The Editors have selected this article as a highlight of the issue.

## Introduction

Inflammation is critical to protect against infection and maintain homeostasis. Macrophages are crucial components of the innate immune system required for mediating the magnitude and outcome of the inflammatory response.^1–3^ By illuminating the mechanisms through which macrophages regulate this response, we will be better equipped to combat infection and inflammatory diseases. Macrophages sense danger signals through PRRs, such as TLR4, which recognizes LPS, triggering complex signaling cascades culminating in the production of pro-inflammatory cytokines.^4–8^ Due to the complex nature of these responses, the steps in inflammatory gene expression are subject to regulation at the level of transcription, pre-mRNA splicing, RNA modification, RNA export, and translation.^9^^,^^10^ In recent years a small number of studies have been carried out to investigate the role that HNRNP proteins play in regulating innate immune responses. It is well-documented that HNRNPs represent a ubiquitously expressed class of proteins that are critical for RNA processing and yet some members of the family play highly specific roles in certain cell types.^11^ For example, HNRNPM regulates gene expression through repression of splicing in macrophages. Repression is only relieved on target genes such as *Il6* following phosphorylation of HNRNPM.^12^ HNRNPU has been reported to translocate from the nucleus to the cytosol following LPS stimulation in macrophages leading to the stabilization of target mRNAs including *Tnf* and *Il6.*^13^ HNRNPA0 has also been implicated in regulating mRNA within the cytosol of macrophages following LPS stimulation, by interacting with AU rich elements in target genes such as *Tnf* influencing their expression.^14^

HNRNPA2B1 is a highly conserved, abundant member of the HNRNPA/B subfamily which is involved in all aspects of RNA metabolism from biogenesis to degradation (eg processing and splicing, trafficking and mRNA translation and stability).^15^^,^^16^ HNRNPA2B1 is localized to the nucleus allowing it to mediate gene expression regulation through its control over transcription initiation, alternative splicing, and RNA export.^16–29^ HNRNPA2B1 plays a central role in RNA metabolism and dysregulation, and mutations in this protein are associated with a range of metabolic and neurodegenerative diseases as well as cancers.^20^^,^^30–34^ Recently HNRNPA2B1 has been reported to function as a DNA sensor within the nucleus facilitating Interferon (IFN) signaling downstream of HSV-1 infection.^17^ Importantly, loss of tolerance to HNRNPA2B1 is recognized as a hallmark of several systemic autoimmune rheumatic diseases (SARDs) such as systemic lupus erythematosus (SLE) and rheumatoid arthritis (RA)^35–39^

While our group and others have previously shown that HNRNPA2B1 can play a role in regulating the innate immune response downstream of TLR signaling ex vivo,^18^^,^^40^ we do not understand the mechanisms by which HNRNPA2B1 regulates gene expression in vivo. To answer this question, we generated a conditional mouse and depleted HNRNPA2B1 in myeloid cells by crossing to LysM-Cre. Compared to control (CTL) mice (HNRNPA2B1^fl/fl^), HNRNPA2B1-deficient (knockout [KO]) mice (HNRNPA2B1^fl/fl^ LysM*-*Cre^+/+^) were more resistant in an endotoxic shock model owing to reduced levels of cytokine production even though the KO mice displayed increased levels of circulating macrophages and neutrophils. In response to LPS ex vivo, HNRNPA2B1-deficient macrophages failed to express many type I and type II IFN family cytokines. At the level of splicing, we detected an increase in the abundance of nonproductive transcript isoforms for several key IFN-stimulated genes, as well as the IFN gamma receptor. Collectively our work demonstrates a key role for HNRNPA2B1 in mounting balanced inflammatory responses in macrophages ex vivo as well as in mouse models of endotoxic shock and infection.

### Materials and methods

#### Mice

Heterozygous floxed mice for the HNRNPA2B1 locus were generated using CRISPR to insert 2 loxP sites flanking exons 2 and 7 in the HNRNPA2B1 locus. Mice homozygous for the loxP sites were used as controls in these experiments. Heterozygous loxP mice were generated by Biocytogen where CRISPR/Cas9 was used to insert 2 loxP fragments in introns 1 and 7 of the HNRNPA2B1 locus. When exons 2 to 7 are removed a protein reading frame shift will occur which results in the production of a 100aa protein that eventually undergoes nonsense-mediated decay (NMD). The sgRNAs and target sequences used to generate the mice are outlined in Table 1 in addition to the genotyping primers in Table 2.

**Table 1.** CRISPR/Cas9 sgRNA utilized to generate HNRNPA2B1-conditional mice.

sgRNA ID	Sequence name	Sequence (5ʹ–3ʹ)
EGE-ZY-021-B-T7-sgRNA6-A	**target sequence**	GTCTTCATACCGTTTCGAGG **TGG**
	EGE-ZY-021-B-T7-sg6-A-dn	CTATTTCTAGCTCTAAAACCCTCGAAACGGTATGAAGACCTATAGTGAGTCGTATTA
EGE-ZY-021-B-T7-sgRNA10	**target sequence**	AGTAATTGGTAACAAGCTGC **AGG**
	EGE-ZY-021-B-T7-sg10-dn	CTATTTCTAGCTCTAAAACGCAGCTTGTTACCAATTACCTATAGTGAGTCGTATTA

The highlighted TGG and AAG represent the protospacer adjacent motif (PAM).

**Table 2.** loxP site integration detection primers.

Primer	Sequence (5ʹ–3ʹ)	Tm (°C)	**Product** **size (bp)**
EGE-ZY-021-B-5ʹloxP-F1	CCGGATTTGGCGGCCGCCATTTTC	60	WT: 342 Mutant: 433
EGE-ZY-021-B-5ʹloxP-R1	CCAGGCCTCGGTTGTACTACGTTC	60	
EGE-ZY-021-B-3ʹloxP-F2	GCATAGGCCTGAGCTCTCAGCATTCTG	58	WT: 573 Mutant: 664
EGE-ZY-021-B-3ʹloxP-R1	GTTGATTTGTTGGGGACATTGAGGG	52	

LysM-Cre mice were purchased from the Jackson Laboratory (Bar Harbor, Maine), reference ID: RRID:IMSR_JAX:004781. Conditional KO mice were homozygous for both the loxP sites and LysM-Cre site. All mouse strains were bred at the University of California, Santa Cruz (UCSC) and maintained under specific pathogen-free conditions in the animal facilities of UCSC. All protocols were performed in accordance with the guidelines set forth by UCSC and Texas A&M Institutional Animal Care and Use Committees.

#### Cell culture

Bone marrow–derived macrophages (BMDMs) were generated by culturing erythrocyte-depleted BM cells in DMEM supplemented with 10% FCS, 5 mL pen/strep (100×), 500 μL ciprofloxacin (10 mg/mL), and 10% L929 supernatant for 7 to 14 d, with the replacement of culture medium every 2 to 3 d.

#### Ex vivo stimulation of macrophages and inflammasome activation

Bone marrow–derived macrophage cells were stimulated with LPS at 200 ng/ml (TLR4) for 5 h for RNA extraction and 18 h for ELISA and Western blots. For inflammasome activation assay, cells were primed with either LPS at 20 ng/mL or IFNγ at 50 ng/mL overnight followed by LPS for 3 h. Inflammasome was activated by ATP at 5 mM for 2 h or dA:dT at 1 μg/mL for 6 h or Nigericin at 25 μM for 0.5 h. For RNA and protein isolation, 1 to 2 × 10^6^cells were seeded in 12-well format or 10 × 10^6^ cells were seeded in 10-cm plates.

Antibodies used for apoptosis assessment: Annexin V: BioLegend 640920 and PI.

#### RNA isolation, cDNA synthesis, and RT-PCR

Total RNA was purified from cells or tissues using Direct-zol RNA MiniPrep Kit (Zymo Research, R2072) and TRIzol reagent (Ambion, T9424) according to the manufacturer’s instructions. RNA was quantified and assessed for purity using a nanodrop spectrometer (Thermo Fisher). Equal amounts of RNA (500 to 1,000 ng) were reverse transcribed using iScript Reverse Transcription Supermix (Bio-Rad, 1708841), followed by qPCR using iQ SYBR Green Supermix reagent (Bio-Rad, 1725122) with the following parameters: 95 °C for 10 min, followed by 40 cycles of 95 °C for 15 s, 60 °C for 30 s, and 72 °C for 30 s, followed by melt-curve analysis to control for nonspecific PCR amplifications. Oligos used in qPCR analysis were designed using Primer3 Input version 0.4.0.

Gene expression levels were normalized to *Actin* or *Hprt* as housekeeping genes.

#### Cells supernatant collection for ELISA

Supernatant was collected from cultured BMDMs, centrifuged at 12000 × *g*, 5 min at room temperature and submitted for cytokine analysis.

#### Serum harvest

Mice were humanely sacrificed; blood was collected immediately postmortem by cardiac puncture. Blood was allowed to clot and centrifuged, serum was stored at −70 °C, then sent to EVE Technologies for measurements of cytokines/chemokines.

#### Spleen tissue harvesting for cytokine measurement

Mice were humanely sacrificed, and their spleens were excised. The whole spleens were snap frozen and homogenized, and the resulting homogenates were incubated on ice for 30 min and then centrifuged at 300 × *g* for 20 min. The supernatants were harvested, passed through a 0.45-µm–pore-size filter, and used immediately or stored at −70 °C, then sent to EVE Technologies for measurement of cytokines/chemokines.

#### RNA sequencing libraries

RNA-Seq was performed in BMDMs with no treatment or treated with LPS for 5 h. The data are accessible at the National Center for Biotechnology Information (NCBI) Gene Expression Omnibus (GEO) database, accession: GSE243269 (https://www.ncbi.nlm.nih.gov/geo/query/acc.cgi? acc=GSE243269).

RNA-Seq was performed in biological triplicates in fl/fl BMDMs used as control and KO BMDMs at 0 and 5 h after LPS treatment (200 ng/mL). RNA-Seq libraries were generated from total RNA (1 μg) using the Bioo kit, quality was assessed, and samples were read on a High-SEq 4000 as paired-end 150-bp reads. Sequencing reads were aligned to the mouse genome (assembly GRCm38/mm10) using STAR. Differential gene-expression analyses were conducted using DESeq2. GO enrichment analysis was performed using PANTHER. Data was submitted to GEO, accession: GSE243269 (https://www.ncbi.nlm.nih.gov/geo/query/acc.cgi? acc=GSE243269).

#### Alternative splicing analysis

Alignment files from RNA-Seq analysis were filtered to include only canonical chromosomes and were passed to Isoform Usage Two-step Analysis (IUTA) to test differential isoform usage.^41^ Gene isoforms from the Gencode M25 Comprehensive gene annotation file were used. Family wise error rate (FWER) was accounted for with Bonferroni correction, and changes were called significant at FWER < 0.01.

#### Cell extracts and Western blots

Cell lysates were prepared in RIPA buffer (150 mM NaCl, 1.0% Nonidet P-40, 0.5% sodium deoxycholate, 0.1% SDS, 50 mM Tris-HCl [pH 7.4], and 1.0 mM EDTA) containing protease-inhibitor mixture (Roche, 5892791001) and quantified using Pierce Bicinchoninic Acid Assay (Thermo Fisher, 23225). When indicated, the NEPER kit (Thermo Fisher Scientific, 78833) supplemented with protease inhibitor mixture (Roche) or 100 U/mL SUPERase-In (Ambion, AM2694) was used for cellular fractionation prior to Western blotting. Equivalent amounts (15 μg) of each sample were resolved by SDS-PAGE and transferred to polyvinylidene difluoride membranes using Trans-Blot Turbo Transfer System (Bio-Rad). Membranes were blocked with PBS, supplemented with 5% (w/v) nonfat dry milk for 1 h, and probed with primary antibody overnight with either HNRNPA2B1 (1:1000, Santa Cruz Biotechnology, sc-374053). Horseradish peroxidase–conjugated β-actin (1:500, Santa Cruz Biotechnology, sc-47778), HNRNPL (1:1000, Santa Cruz Biotechnology, sc-32317), or GAPDH (1:1000, Santa Cruz Biotechnology, sc-32233) was used as loading control. Horseradish peroxidase-conjugated goat anti-mouse (1:10,000, Bio-Rad, #1721011) or anti-rabbit (1:2,000, Bio-Rad, #1706515) secondary antibodies were used. Western blots were developed using Amersham enhanced chemiluminescence (ECL) Prime chemiluminescent substrate (GE Healthcare, 45-002-401) or Pierce ECL (Life Technologies, 32106).

#### In vivo LPS-Induced endotoxic shock assay

Age- and sex-matched CTL and KO mice (10 to 12 wk old) were i.p. injected with PBS as a control or *E. coli* LPS (5 mg/kg/animal). For gene expression and cytokine analysis, mice were euthanized 6 h or 18 h postinjection. Blood was collected immediately postmortem by cardiac puncture. Serum was submitted to Eve Technologies for cytokine analysis.

#### Assessment of immune cell populations using flow cytometry

Blood was collected immediately postmortem by cardiac puncture, and single-cell suspensions prepared from the spleen of CTL and HNRNPA2B1 KO mice were depleted of RBCs prior to staining. Whole spleens were collected, homogenized, and depleted of RBCs.

Fragment crystallizable (Fc) receptors were blocked (anti-CD16/32, BD Pharmingen) prior to staining with LIVE/DEAD Fixable Near-IR Dead Cell Stain (Thermo Fisher), anti–CD11b-Alexa Fluor 488, anti–LY6G-BV421, anti–LY6C-PE-cy7, anti–CD19 BV786, anti–CSF-1R APC, anti–CD3-PE, anti–SiglecF Super Bright 645, and anti–F4/80 PE-eFluor 610, MHCII BV421, CD86 Super Bright 645, CD80 PE-Cy7, IFNGRI BV605, IFNGRII PE, all by Thermo Fisher.

Flow cytometry analysis of BMDMs harvested from CTL and HNRNPA2B1 KO mice was performed; cells were stained with LIVE/DEAD Fixable Near-IR Dead Cell Stain (Thermo Fisher), anti–CD11b-Alexa Fluor 488, anti–F4/80 PE-eFluor 610, MHCII BV421, CD86 Super Bright 645, CD80 PE-Cy7, IFNGRI BV605, IFNGRII PE, all by Thermo Fisher.

Data acquisition was performed using Attune NxT (Thermo Fisher). Analysis was performed using FlowJo analysis software (BD Biosciences).

#### Phagocytosis assay

CTL and KO BMDMs were cultured as described above. Cells were then administered with pHrodo Green *E. coli* BioParticles (Invitrogen) conjugates for 0, 15, 30, and 60 min at 37 °C. pHrodo Green fluorescence, a measure of *E. coli* in an acidified phagosome, was examined by flow cytometry.

#### *S. enterica* (ser. Typhimurium)

*S. enterica* serovar Typhimurium (SL1344) was obtained from Dr. Denise Monack, Stanford. The serovar Typhimurium stocks were streaked out on LB agar plates and incubated at 37 °C overnight. For serovar Typhimurium infection, overnight cultures of bacteria were grown in LB broth containing 0.3 M NaCl and grown at 37 °C until they reached an OD600 of 0.9. On the day of infection cultures were diluted 1:20. Once cultures had reached mid-log phase (OD600 0.6 to 0.8) 2 to 3 h, 1 mL of bacteria was pelleted at 5000 rpm for 3 min and washed twice with 2× PBS.

#### In vivo *S. enterica* (ser. Typhimurium) infection

Age- and sex-matched CTL and KO mice (10 to 12 wk old) were i.p. injected with *S. enterica* serovar Typhimurium 2.5 × 10^4^ bugs per animal. For CFUs and cytokine analysis, mice were euthanized 3 days postinfection. Blood was collected immediately postmortem by cardiac puncture. Serum was submitted to Eve Technologies for cytokine analysis. For time to death, mice were monitored for signs of imminent morbidity (eg hunched posture, ruffled coat, lethargy as described in^42^) while blinded to genotype.

#### Ex vivo *S. enterica* infection

Infection of BMDMs with wild-type (WT) *S. enterica* was done as described in.^43^ Briefly, WT *S. enterica*, strain SL1344, was grown in LB media overnight (O/N) at 37 °C and back-diluted once the following morning until it had reached OD600 = 1.0. The bacteria were pelleted at 4,000 rcf for 5 min, washed once with HBSS, pelleted again, and resuspended in HBSS to OD600 = 1.0. This resuspension was then diluted to an MOI: 10 per 250 μL (infection inoculum, 3,000,000 CFUs per 250 μL). Three hundred thousand d-7 BMDMs were seeded per well, in triplicate per genotype/time point/condition in a 24-well non-TC dish in BMDM media (pyruvate supplemented DMEM, 20% FBS [Sigma-Aldrich], 10% M-CSF media). In the evening of d 7, cells were pretreated with 100 ng/mL rmIFN-gamma or sham for 48 h. On the morning of d 10, BMDMs were washed once with HBSS prior to addition of infection inoculum. Infection was “initiated” by a spinfection, 500 rcf for 10 min; afterwards plates were incubated at 37 °C, 5% CO_2_ for 20 min. After 20-min incubation, cells were washed 3× with HBSS before supplementing cells with Hi gent media (BMDM media + 100 μg/mL gentamicin) and incubating at 37 °C + 5% CO_2_, the 0-h time point was collected prior to addition of Hi gent media. After 1 h, Hi gent media was aspirated and replaced with Lo gent media (BMDM media + 15 μg/mL gentamicin), and returned to incubation at 37 °C + 5% CO_2_. Collection of CFUs occurred; at specified time points (0 h, 2 h, 8 h), wells were washed 1× with HBSS prior to addition of lysis buffer (PBS + 1% Triton X-100 + 0.1% SDS) and incubated for 10 min at room temperature. Lysates were then collected and serially diluted (10% per step) out to 10^−6^ in HBSS, vortexing between dilutions. Dilutions from 10^−4^, 10^−5^, and 10^−6^ were plated in duplicate from each well on LB plates and incubated overnight, counted the next morning.

#### Percentage-of-cell-death assessment

To assess cell death during *S. enterica* infection, BMDMs were plated in 96-well half area clear bottom plates (Corning) at 2.5 + 10^4^ cells/well in 50 µL of media using a multichannel pipette. After adherence to plate (approximately 1 to 2 h), an additional 25 µL of media was added to each well containing cells. The following day media was removed and 50 µL DMEM containing *S. enterica* MOI of 2 was added. Bacteria/cells were spun at 400 rcf for 10 min, then incubated for 10 min at 37 °C. Following incubation, infection media containing *S. enterica* was removed and 100 µL complete media (pyruvate-supplemented DMEM, 20% FBS [Sigma], 10% M-CSF media) containing 5 µg/mL PI (Thermo Fisher) was added to each well. Exposure/focus was calibrated at 4× magnification on a Cytation 5 plate reader (BioTek). Total cell numbers were determined using NucBlue (Thermo Fisher) (2 drops per milliliter) in 1× PBS with a subset of the plated cells. For image analysis Gen 3.5 software (BioTek) was used. Dead cells were counted by PI staining of nucleus over a time course.

All reagents and primers utilized in this study are outlined in Table 3.

**Table 3.** Key resources.

Reagent or resource	Source	Identifier
**Antibodies**		
HNRNPA2B1 mouse monoclonal Ab	Santa Cruz Biotechnology	Sc-374053
pSTAT3	Cell Signaling	9138S
STAT3	Cell Signaling	9139S
pJAK3	Cell Signaling	5031S
JAK3	Cell Signaling	8863S
Horseradish peroxidase–conjugated β-actin	Santa Cruz Biotechnology	Sc-47778
HNRNPL	Santa Cruz Biotechnology	Sc-32317
GAPDH	Santa Cruz Biotechnology	Sc-32233
Horseradish peroxidase–conjugated goat anti-mouse	Bio-Rad	#1721011
Horseradish peroxidase–conjugated goat anti-rabbit	Bio-Rad	#1706515
Annexin V	BioLegend	640920
anti-CD16/32	BD Pharmingen	553141
LIVE/DEAD Fixable Near-IR Dead Cell Stain	Thermo Fisher	L34992
anti–CD11b-Alexa Fluor 488	Thermo Fisher	53-0112-82
anti–LY6G-BV421	Thermo Fisher	404-9668-80
anti–LY6C-PE-cy7	Thermo Fisher	25-5932-80
anti–CD19 BV786	Thermo Fisher	417-0193-80
anti–CSF-1R APC	Thermo Fisher	17-1152-80
anti–CD3-PE	Thermo Fisher	12-0031-81
anti–SiglecF Super Bright 645	Thermo Fisher	64-1702-82
anti–F4/80 PE-eFluor 610	Thermo Fisher	61-4801-82
MHCII BV421	Thermo Fisher	404-5321-80
CD86 Super Bright 645	Thermo Fisher	64-0862-80
CD80 PE-Cy7	Thermo Fisher	25-0801-80
IFNGRI BV605	Thermo Fisher	745111
IFNGRII PE	Thermo Fisher	113604
**Chemicals, peptides, and recombinant proteins**		
LPS *E. coli*	Sigma-Aldrich	L2630-25MG
IFNG	BioLegend	575304
ATP	Sigma	A6419
Nigericin	Sigma	N7143-5MG
**Critical commercial assays**		
NEXTflex Rapid Illumina RNA-Seq Library Prep Kit	Bioo Scientific	
Cell Proliferation Kit I (MTT)	MilliporeSigma	11465007001
IL1b Mouse DuoSet ELISA Kit	R&D	DY401
pHrodo Green *E. coli* BioParticles Conjugate for Phagocytosis	Thermo Fisher	P35366
**Oligonucleotides**		
5ʹ loxP_F	CCGGATTTGGCGGCCATTTTC	
5ʹ loxP_R	CCAGGCCTCGGTTGTACTACGTTC	
3ʹ loxP_F	GCATAGGCCTGAGCTCTCAGCATTCTG	
3ʹ loxP_R	GTTGATTTGTTGGGGACATTGAGGG	
Cre_Rxn_A	CCCAGAAATGCCAGATTACG	
Cre_Rxn_B	TTACAGTCGGCCAGGCTGAC	
Cre_Rxn_common	CTTGGGCTGCCAGAATTTCTC	
Irf7 F	GACTGGGAAAGATCACCGGC	
Irf7 R	TTGCGCCAAGACAATTCAGG	
Irf8 F	CAATCAGGAGGTGGATGCTTCC	
Irf8 R	GTTCAGAGCACAGCGTAACCTC	
STAT3 F	AGGAGTCTAACAACGGCAGCCT	
STAT3 R	GTGGTACACCTCAGTCTCGAAG	
Oac1c F	GACTTCCGACATCAAGAGGTCTG	
Oac1c R	ATCCAGGTCTGAGCCTCCTTTG	
Ifi208 F	GCCACTCAAGGCAAAGATAGGATCTC	
Ifi208 R	CTGGGGATTCTGCATTTCATTGTCCTC	

## Results

### HNRNPA2B1-deficient mice display altered immune responses following endotoxic shock in vivo

**Figure 1.**
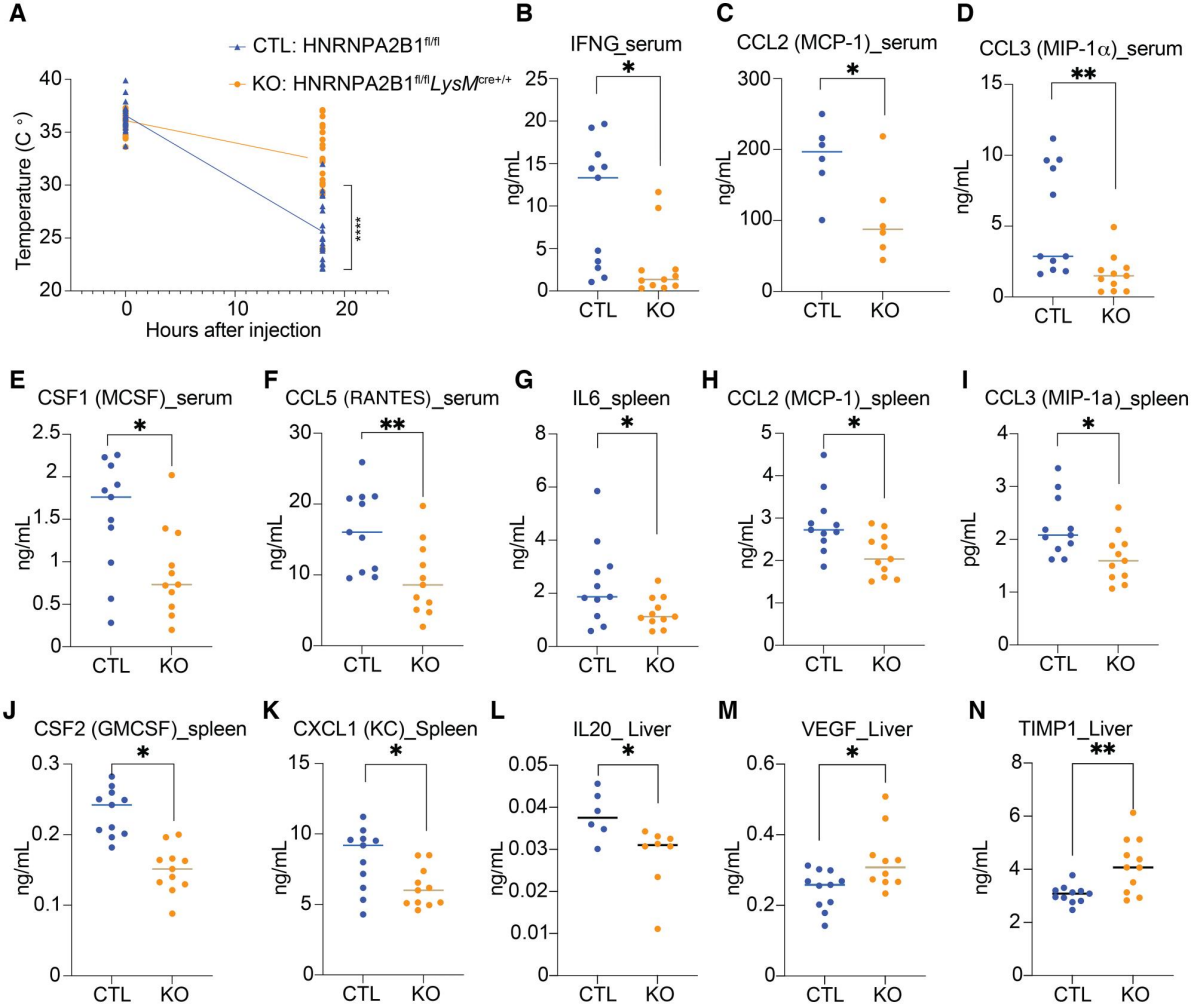
HNRNPA2B1 KO mice show altered immune responses following endotoxic shock in vivo. (A) Temperature change in CTL and HNRNPA2B1 KO mice after i.p. injection with 5 mg/kg LPS. N = 25 CTL, 26 KO. (B–N) Cytokine levels in serum, spleen, and liver of mice treated with 5 mg/kg LPS 6 h postinjection. (B) IFNG_serum, (C) CCL2_serum, (D) CCL3_serum, (E) CSF1_serum, (F) CCL5_serum, (G) IL6_spleen, (H)CCL2_spleen, (I) CCL3_spleen, (J) CSF2_spleen, (K) CXCL1_spleen, (L) IL20_liver, (M) VEGF_liver, (N) TIMP1_liver. Student’s *t* tests were performed using GraphPad Prism. Asterisks indicate significant differences between mouse lines (*P ≤ 0.05, **P ≤ 0.01).

We previously reported that shRNA-mediated knockdown of HNRNPA2B1 impacted genes downstream of NF-κB signaling in macrophages in vitro.^18^ Here we set out to investigate whether specific loss of HNRNPA2B1 in myeloid cells could impact this pathway in vivo. To this end we generated an HNRNPA2B1-conditional KO mouse using an LysM*-*Cre system (Fig. S1A). We inserted 2 loxP sites flanking exons 2 and 7 of the HNRNPA2B1 locus using CRISPR and crossed to LysM-Cre to homozygosity to selectively delete HNRNPA2B1 from myeloid cells. Western blot analysis confirmed complete knockout of HNRNPA2B1 in the deficient BMDMs (Fig. S1D). Mice appeared normal and bred at expected mendelian ratios. Immune populations were profiled at baseline in blood and spleen, and no difference was observed in immune cell numbers for monocytes, macrophages, eosinophils, T or B cells (Fig. S1E and F). A small increase in neutrophils was recorded in the spleen (Fig. S1F). HNRNPA2B1 deletion did not impact macrophage phagocytic function as measured by pHrodo Green *E. coli* particle uptake (Fig. S1G). To determine how HNRNPA2B1-deficient mice respond to endotoxic shock, mice were injected through the intraperitoneal route with 5 mg/kg LPS. One early clinical feature of endotoxic shock in mice is the rapid decrease in body temperature. Interestingly, HNRNPA2B1-deficient mice showed a much lower decrease (32 °C) in temperature compared to WT (∼25 °C) following LPS administration for 18 h (Fig.1, A). Cytokine levels were reduced in the serum, spleen, and liver of the KO mice compared to CTL (Fig.1, B–N). CCL2 and CCL3 were reduced in both serum and spleen (Fig.1C, D, H, and I), while other cytokines including IFNG, CSF1, and CCL5 were reduced only in the serum (Fig. 1B, E. and F) or restricted to the spleen (IL6, CSF2, and CXCL1) (Fig.1G, J, and K). Only IL20 was reduced in the KO livers compared to CTL while VEGF and TIMP1 were increased (Fig. 1M and N). No difference in IFNB levels was measured in lung, liver, or spleen (Fig. S2). From these data we can conclude that specific loss of HNRNPA2B1 from myeloid cells resulted in significant changes in the pro-inflammatory cytokine levels throughout tissues of the knockout mice, indicating that HNRNPA2B1 plays an important and specific role in regulating pro-inflammatory gene expression.

### Macrophages are elevated in the HNRNPA2B1-deficient mice and display altered costimulatory molecule expression

**Figure 2.**
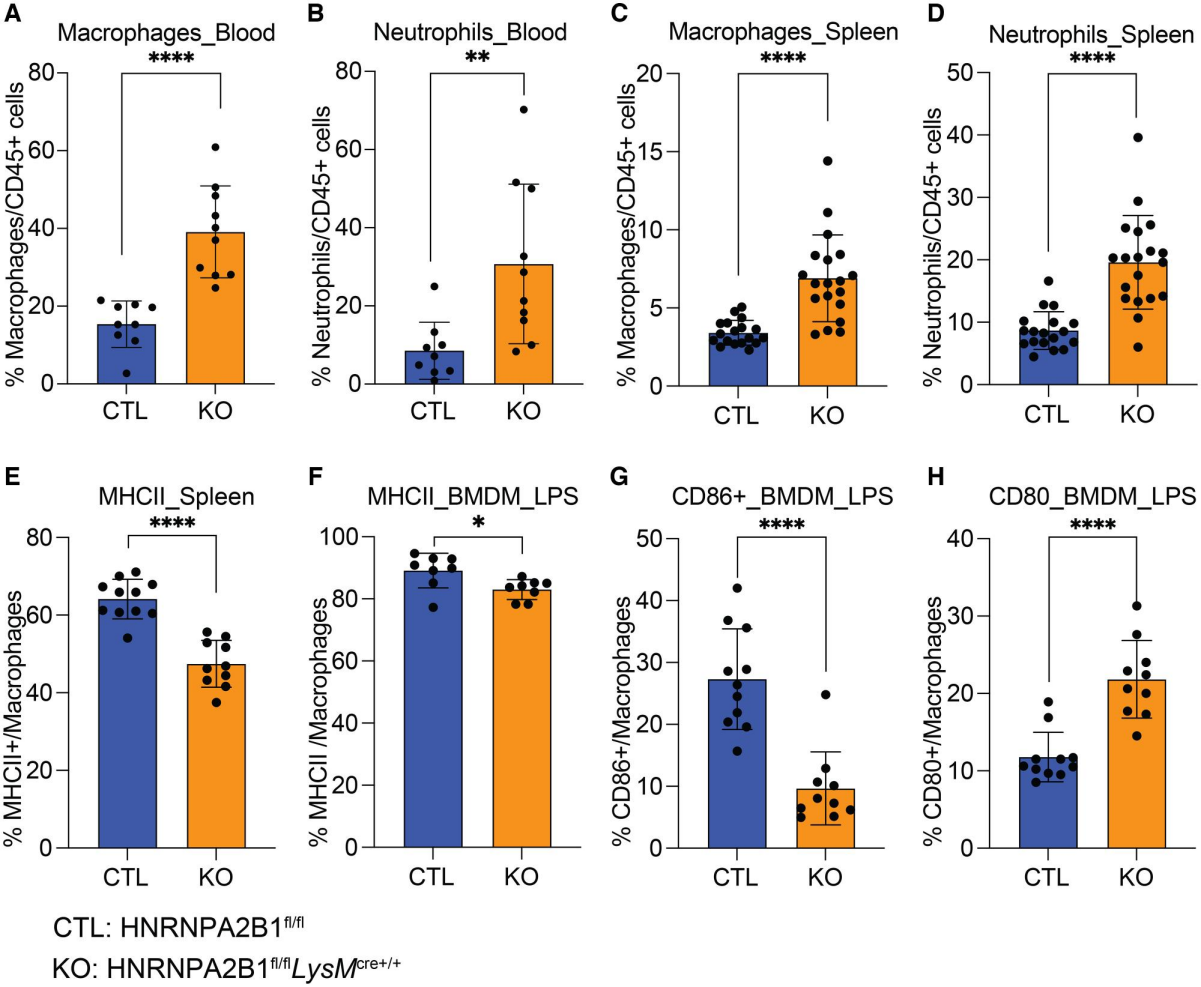
Macrophage and neutrophil levels are elevated in the HNRNPA2B1 KO mice and show altered macrophage activation following endotoxic shock. (A) Macrophage levels from CTL and HNRNPA2B1 KO mice blood, measured using a flow cytometry panel. (B) Neutrophil levels from CTL and HNRNPA2B1 KO mice blood, measured using a flow cytometry panel. (C) Macrophage levels from CTL and HNRNPA2B1 KO mice spleen, measured using a flow cytometry panel. (D) Neutrophil levels from CTL and HNRNPA2B1 KO mice spleen, measured using a flow cytometry panel. (E) Level of MHCII marker on macrophage surface in the spleen; analysis performed using flow cytometry. (F–H) Level of activation markers on BMDMs ex vivo; analysis performed using flow cytometry: (F) MHCII, (G) CD86, (H) CD80. Student’s *t* tests were performed using GraphPad Prism. Asterisks indicate significant differences between mouse lines (*P ≤ 0.05, **P ≤ 0.01).

Since HNRNPA2B1 is a known viability gene,^44^ we speculated that downregulated cytokine response could be simply due to fewer macrophages and neutrophils in the deficient mice. Interestingly we found the opposite to be true, with the KOs showing increased macrophage and neutrophil counts in the spleen and blood following endotoxic shock (Fig. 2A–D). There was a small increase in T cells in the KO spleens but not the blood, and all other immune cells including B cells, monocytes, and eosinophils were similar comparing KO to CTL (Fig. S3A–H). We also investigated whether peritoneal macrophages’ (PMs’) numbers increased since they are directly exposed to LPS upon injection, but no changes in PM levels post–LPS introduction were observed (Fig. S3I and J).

Following stimulation, macrophages upregulate several activation markers including costimulatory molecules CD86, CD80, and MHC Class II. We found that MHC Class II expression was reduced in the spleens and isolated BMDMs from HNRNPA2B1-deficient mice (Fig. 2E and F). In addition, although CD86 levels were reduced, CD80 levels were higher in BMDMs following LPS stimulation (Fig. 2G and H). From these data we can conclude that although macrophages are more abundant in HNRNPA2B1 KO LPS-exposed mice, they display attenuated responses following inflammatory activation.

### HNRNPA2B1-deficient mice are susceptible to *S. enterica* infection

**Figure 3.**
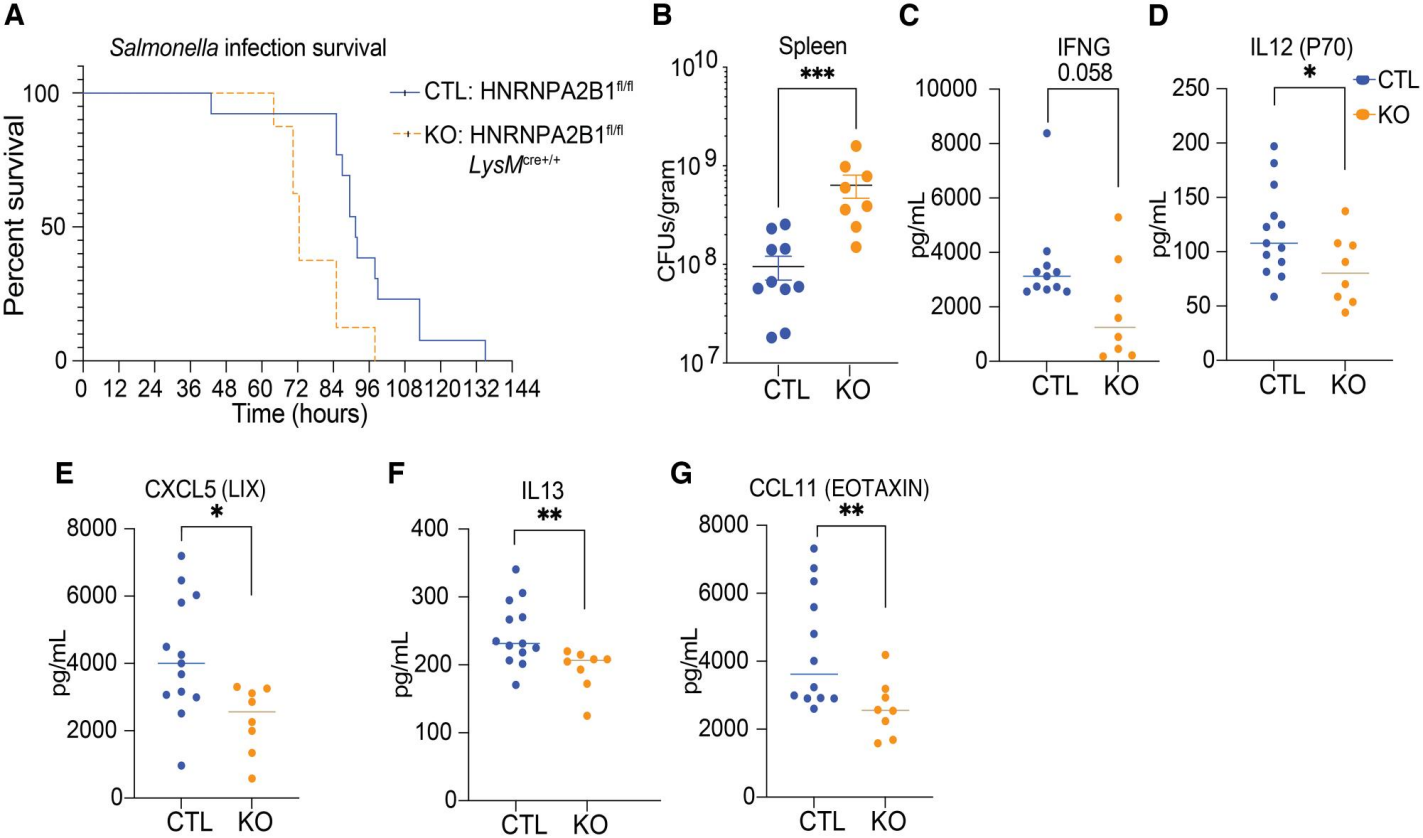
HNRNPA2B1 KO mice are susceptible to *Salmonella* infection. (A) Survival of CTL and HNRNPA2B1 KO mice after i.p. *Salmonella* infection. (B) CFU measurement of *Salmonella* bacterial load in mouse spleen. (C–G) Cytokine levels in serum of CTL and KO mice infected with *Salmonella* were measured by Elisa, (C) IFNG, (D) IL12 (P70), (E) CXCL5, (F) IL13, (G) CCL11.

Given the altered inflammatory responses observed in the HNRNPA2B1-deficient mice during endotoxic shock, we wanted to investigate the role of HNRNPA2B1 in controlling the innate immune response to pathogens. To this end, we chose *S. enterica* serovar Typhimurium as it utilizes macrophages as a replicative niche and is controlled in macrophages via IFNG.^45^ We infected CTL and HNRNPA2B1 KO mice by i.p. delivery of *Salmonella* (2.5 × 10^4^) and assessed for signs of imminent morbidity (eg hunched posture, ruffled coat, lethargy as described in^42^) over the course of 6 d. HNRNPA2B1-deficient mice succumbed to infection earlier than controls (Fig. 3A). They experienced higher bacterial burdens in the spleen (Fig. 3B) and lower circulating cytokines including IFNG, IL12, IL13, CXCL5, and CCL11 (Fig. 3C–G).

### HNRNPA2B1-deficient macrophages have altered gene expression at steady state and following inflammation

**Figure 4.**
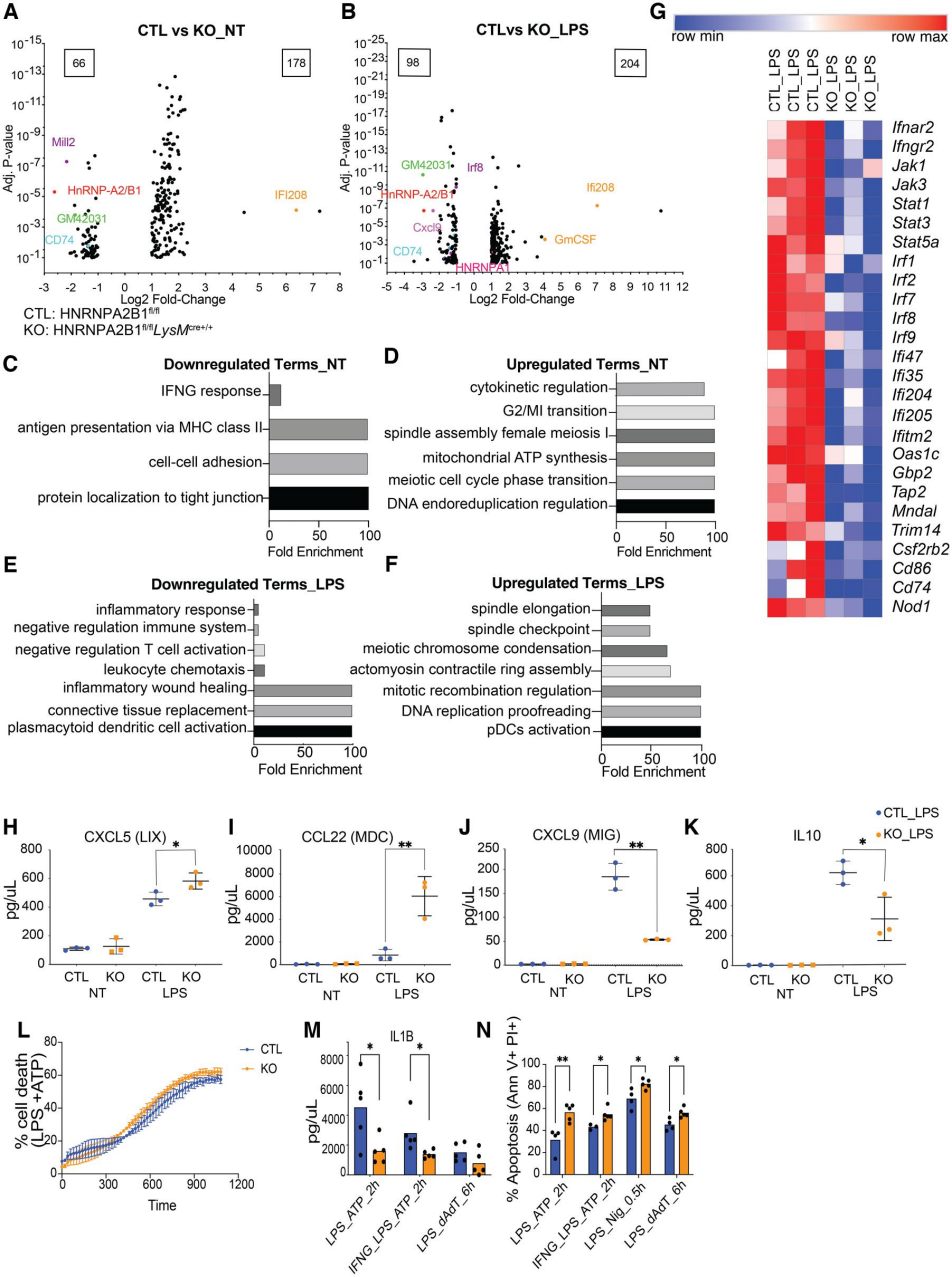
HNRNPA2B1 KO macrophages have altered IFN signaling in response to LPS, and KO macrophages fail to clear the pathogen and switch to an alternate form of cell death. (A) Volcano plot of differentially expressed (DE) genes in HNRNPA2B1 KO BMDMs at baseline. (B) Volcano plot of DE genes in HNRNPA2B1 KO BMDMs under LPS stimulation. (C) Gene ontology analysis of downregulated genes at baseline. (D) Gene ontology analysis of upregulated genes at baseline. (E) Gene ontology analysis of downregulated genes post–LPS stimulation. (F) Gene ontology analysis of upregulated genes post–LPS stimulation. (G) Heat map of IFNG response genes’ normalized counts in CTL and KO cells post–LPS stimulation. (H–K) Cytokine levels as measured by ELISA from BMDM supernatant. The supernatant was harvested from CTL and KO cultured BMDMs; and multiplex cytokine analysis was performed for (H) CXCL5 (LIX), (I) CCL22 (MDC), (J) CXCL9 (MIG), (K) IL10. Each dot represents BMDMs from an individual animal. Error bars represent the standard deviation of biological triplicates. Student’s *t* tests were performed using GraphPad Prism. Asterisks indicate significant differences (*P ≤ 0.05, **P ≤ 0.01). (L) Bacterial growth in ex vivo–infected BMDMs over 8-h time course comparing HNRNPA2B1 KO to Cre CTLs. (I) Rate of total cell death in BMDMs under inflammasome activation. (M) Levels of released IL1b in CTL and KO macrophages under inflammasome activation. (N) Levels of apoptosis markers in BMDMs under inflammasome activation. Student’s *t* tests were performed using GraphPad Prism. Asterisks indicate significant differences between mouse lines (*P ≤ 0.05, **P ≤ 0.01).

To gain mechanistic insights into the specific defects occurring in the HNRNPA2B1-deficient macrophages, we performed RNA sequencing (RNA-Seq) comparing CTL and KO BMDMs at baseline and following 5-h LPS stimulation. One hundred seventy-eight genes were upregulated while 66 were downregulated in the HNRNPA2B1-deficient cells compared to control at baseline using a log 2-fold-change cutoff of 1, –1 and an adjusted *P* value of less than 0.1 (Fig. 4A and B, Table S1 and GSE243269). Gene ontology (GO term) analysis of differentially expressed (DE) genes showed an overall downregulation of inflammatory response genes such as *Cd74* and *Mill2* at baseline, as well as genes such as *Irf8* and *Cxcl9* after LPS treatment in the deficient cells (Fig. 4A–C and E). Upregulated GO-terms at baseline consisted mostly of genes involved in cell cycle and cell division regulation as well as DNA replication (Fig. 4D and F). There was a strong enrichment of IFN response genes including receptors (*Ifnar2*, *Ifngr2*), signaling molecules (*Jaks*, *Stats*), and transcription factors (*Irf1*, *2*, *7*, *8*, *9*) that were all downregulated in the HNRNPA2B1-deficient macrophages (Fig. 4G). We further identified the specificity of the downregulated IFN response genes by performing IFN subtyping analysis using the Interferome database.^46^^,^^47^ Consistent with a type II IFN defect, the downregulated genes were identified as type II IFN or type I/II IFN response genes (Fig. S4). Using RT-PCR, we confirmed decreased expression levels of *Irf7*, *Irf8*, *Stat3*, and *Oas1c* and an increase in *Ifi208* in the deficient cells (Fig. S5A–E).

To determine if the effects of HNRNPA2B1 loss extends beyond RNA levels, we utilized a multiplex ELISA and measured altered expression of many key inflammatory cytokines in HNRNPA2B1 KO BMDMs, with some showing increased expression (CXCL5, CCL22, CSF1, CSF3, CCL17, IL1B, IL5, IL12, and IL15), others showing decreased expression (eg CXCL9, IL10, and TIMP1) poststimulation (Fig. 4H–K, Fig. S6A–H), and a number of proteins remained unchanged (Fig. S6I–M). Total protein levels of STAT1, 3, and IRF7 were not impacted in the knockout cells as measured by Western blot (Fig. S7A–C). Collectively, our data indicates that HNRNPA2B1 plays an important role in regulating distinct immune gene expression at the RNA and protein levels.

Given the altered ability of HNRNPA2B1-deficient macrophages to respond to LPS, we next hypothesized that they may be impaired in their ability to respond to pyroptotic triggers, as PAMP sensing commonly serves as “Signal 1” to prime the inflammasome.^5^ To begin to determine whether HNRNPA2B1 plays a role in macrophage cell death, we treated BMDMs with LPS + ATP to activate the NLRP3 inflammasome and measured propidium iodide incorporation over time. We observed no major differences in total cell death between WT and HNRNPA2B1 KO macrophages. Despite overall cell death being similar (Fig. 4L), we found that HNRNPA2B1-deficient macrophages release less IL1B (Fig. 4M), suggesting a defect in either priming or activating the inflammasome. Concomitant with a failure to undergo canonical pyroptosis, HNRNPA2B1-deficient macrophages are more prone to undergo inflammasome-mediated apoptosis, as measured by annexin V+ propidium iodide (PI)+ cells (Fig. 4N). While the effects are modest, together these findings suggest that when HNRNPA2B1-deficient macrophages face an inflammasome trigger, they are more prone to undergo apoptosis than pyroptosis. This may affect the ability of HNRNPA2B1-deficient macrophages to respond to and clear *Salmonella*-infected cells in vivo.

### HNRNPA2B1 regulates specific immune genes through alternative splicing

**Figure 5.**
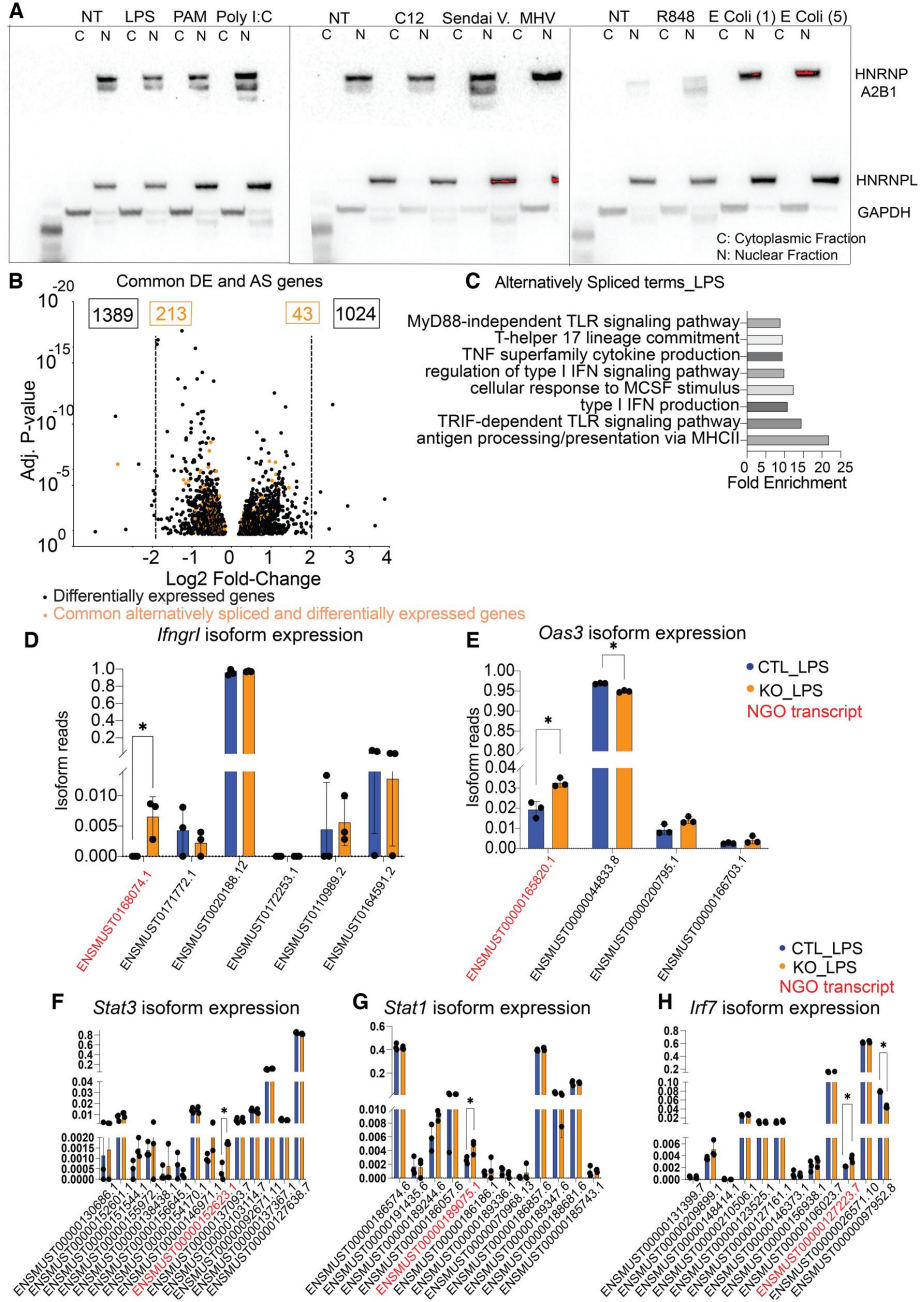
HNRNPA2B1 regulates IFNG signaling through alternative splicing. (A) CTL BMDM nuclear and cytoplasmic fractions were analyzed using Western blots after treatment with a panel of immune stimuli to assess changes in HNRNPA2B1 localization. (B) Volcano plot of DE genes (black) and common DE and alternatively spliced genes in orange in KO BMDMs. (C) Gene ontology analysis of genes that are differentially expressed as well as alternatively spliced in KO BMDMs. (D–H) Isoform usage levels analyzed by IUTA showing switching in isoform usage between CTL and KO BMDMs in (D) *Ifngr1*, (E) *Oas3*, (F) *Stat3*, (G) *Stat1*, (H) *Irf7*. Student’s *t* tests were performed using GraphPad Prism. Asterisks indicate significant differences between mouse lines (*P ≤ 0.05, **P ≤ 0.01).

HNRNPA2B1 is a well-recognized regulator of alternative splicing.^24–27^ Having confirmed strict nuclear localization of HNRNPA2B1 under a variety of inflammatory stimuli (Fig.5A, Fig. S8A), we hypothesized that it regulates IFN response genes through alternative splicing. We employed IUTA^41^ to detect differential usage of gene isoforms in the KO BMDMs after LPS treatment. We found that only a small portion of the downregulated (DE) genes were alternatively spliced (213 out of 1389) in the KO macrophage. Likewise, only 43 out of 1024 upregulated DE genes were alternatively spliced in HNRNPA2B1 KO macrophages (Fig.5B). GO-term analysis revealed that several downregulated alternatively spliced genes were involved in the IFN response (Fig.5C). Taking into consideration the changes in IFN pathway genes’ expression in the KO BMDMs (Fig. 4G) we speculated that these changes are modulated by alternative splicing events. We performed IUTA analysis, which revealed differential isoform expression in the *IfngrI*, *Stat3*, *Stat1*, *Irf7*, and *Oas3* (Fig. 5D–H) loci as a result of HNRNPA2B1 deletion, leading to increased expression of NGO transcripts, which we identified using FLAIR,^48^ which lack a start codon and therefore are not translated (Fig.5D –H). Our results indicate that HNRNPA2B1 regulates specific innate immune genes through splicing.

### HNRNPA2B1 regulates cell surface expression of the IFNG receptor (IFNGR)

**Figure 6.**
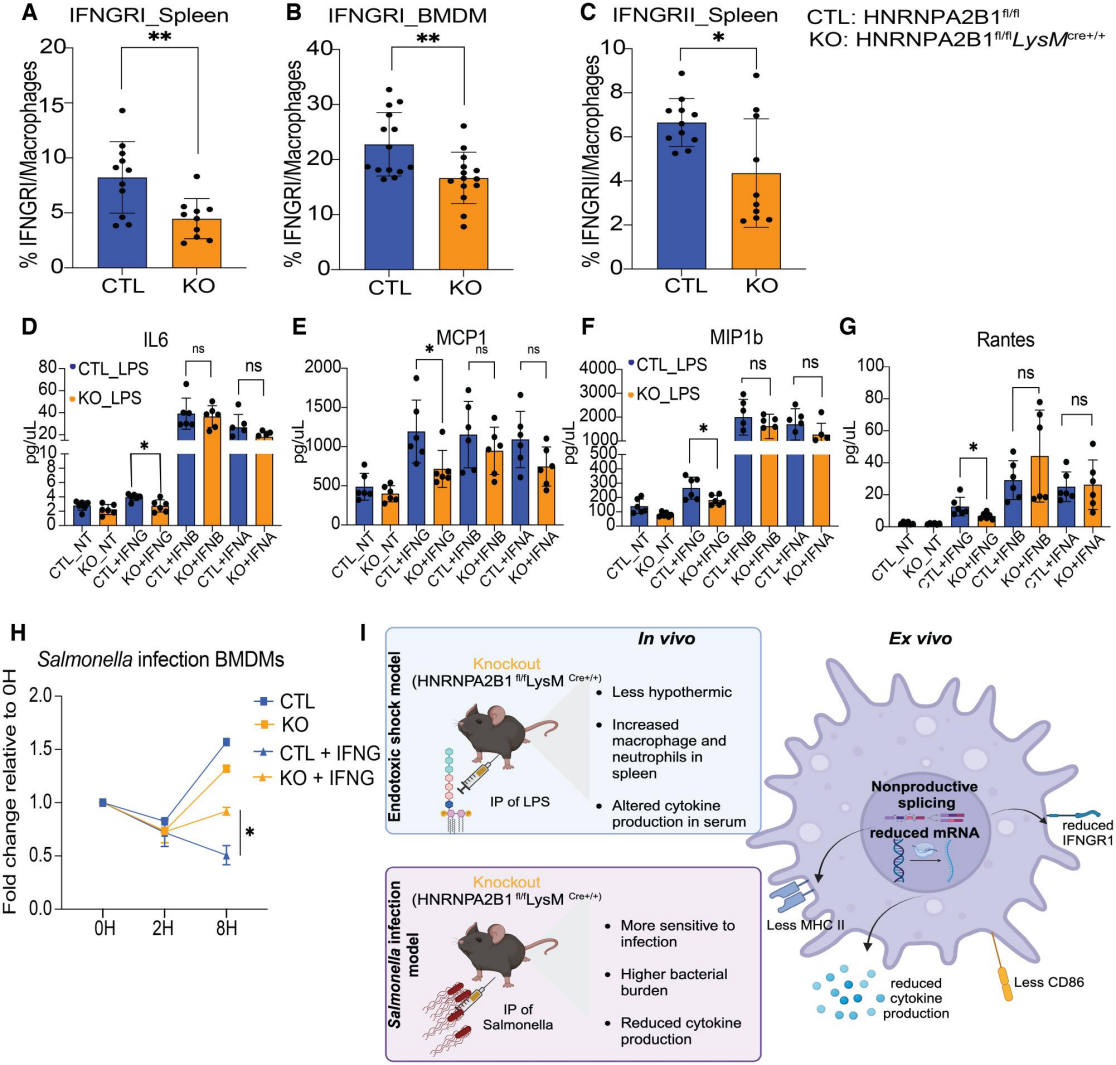
HNRNPA2B1 regulates alternative splicing of IFN response genes. (A) IFNGRI levels on macrophages in the spleen was measured using flow cytometry. (B) IFNGRI levels on macrophages was measured ex vivo using flow cytometry. (C) IFNGRII levels on macrophages in the spleen was measured using flow cytometry. (D–G) BMDMs from CTL and KO mice were stimulated with IFNG, IFNB, or IFNA for 24 h. Supernatants were removed and ELISAs were performed to measure (D) IL6, (E) MCP1, (F) MIP1b, and (G) RANTES. Student’s *t* tests were performed using GraphPad Prism. Asterisks indicate significant differences between mouse lines (*P ≤ 0.05, **P ≤ 0.01) (H) Bacterial growth in *ex vivo*–infected BMDMs over 8 h time course comparing HNRNPA2B1 KO to Cre CTLs. (I) Schematic outlining impacts of HNRNPA2B1 knockout in vivo.

Given the decreased *Ifngr* gene expression and nonproductive spliced isoforms in KO BMDMs (Fig. 4G), along with reduced IFNG production in KO mice (Fig.1, B), we hypothesized that IFNGR protein expression on the macrophage cell surface might be diminished. To this end we quantified both IFNGRI and II levels in macrophages from mice post–LPS injection (Fig. 6A and C, gating strategy in Fig. S9), and on BMDMs treated with LPS ex vivo (Fig. 6B, gating strategy in Fig. S10), and confirmed lower levels of the receptors on the cell surface. To investigate whether this impairment in cell surface expression of the IFNGR could impact downstream responses, we treated CTL and KO BMDMs with exogenous IFNG or with type 1 IFNs to assess changes in cytokine release. KO macrophages were less responsive to IFNG but responded similarly to WT when treated with IFNA or IFNB, indicating a specific defect in the IFNG signaling cascade. Finally, we tested if impaired responsiveness to IFNG could impact the ability of macrophages to control *Salmonella* replication. Next, to determine if HNRNPA2B1-deficient macrophages were defective for cell intrinsic control of *Salmonella* replication, we performed an ex vivo infection of macrophages. Overall, we saw that KO macrophages responded weakly to exogenous IFNG treatment compared to CTL cells (Fig. 6H), resulting in reduced IFNG-mediated killing in the absence of HNRNPA2B1 at 8 h postinfection. This result provides further support for our model whereby the failure to properly splice *Ifngr1* transcripts in HNRNPA2B1-deficient macrophages limits their ability to restrict bacterial replication following IFNG treatment. Fig.6I provides a schematic summary of the observed in vivo phenotypes of the HNRNPA2B1-deficient mice and associated mechanisms. While HNRNPA2B1 can clearly impact many cellular processes, here we highlight its specificity in regulating immune genes in macrophages. Its involvement in splicing of the IFNG receptor is of note given the importance of this signaling cascade in protection against intracellular pathogens like *Salmonella* in macrophages in vivo.

## Discussion

Here we show that HNRNPA2B1 plays roles in regulating immune gene expression in macrophages. We previously reported that HNRNPA2B1 regulates immune gene expression using shRNAs in macrophages ex vivo,^18^ where HNRNPA2B1 knockdown led to increased expression of a subset of IFN response genes. Here we performed RNA-Seq analysis comparing HNRNPA2B1-deficient cells (KOs) to CTL. Consistent with our earlier findings using shRNA-mediated silencing of HNRNPA2B1, we observed widespread changes in the macrophage transcriptome. However, the effects we observed in the HNRNPA2B1 knockouts were more pronounced compared to the shRNA results likely owing to more efficient removal of the gene and the use of primary macrophages rather than immortalized cells. At steady state there were nearly 3 times as many genes upregulated in the deficient cells than downregulated. IFI208, CXCL5, and CCL22 were all confirmed to be upregulated, suggesting that for many genes, HNRNPA2B1 acts as a negative regulator. Further research is needed to elucidate the exact mechanisms by which HNRNPA2B1 represses immune genes, specifically whether its effects are mediated through transcriptional or splicing processes.

HNRNPA2B1-deficient mice showed opposite effects in vivo in response to LPS-induced endotoxic shock versus *Salmonella* infection (Fig.1 and Fig. 3). The KOs are more resistant to LPS yet more sensitive to *Salmonella* infection compared to CTLs. Mice deficient in HNRNPA2B1 produced less proinflammatory cytokines including CCL2 (MCP1), CCL3 (MIP1a), CCL5 (RANTES), CXCL1 (KC), and IL6 in serum and spleen. This is consistent with observations from a previous study examining haploinsufficiency in HNRNPA2B1 mice, which displayed downregulated gene expression.^40^ The disruption in the inflammatory response we observed proved advantageous to KO mice during endotoxic shock as their clinical symptoms including body temperature were elevated compared to controls. While we initially hypothesized that lower cytokine expression in vivo could be explained by reduced myeloid cell numbers given HNRNPA2B1 essential functions in the cell,^18^^,^^24–28^^,^^49–51^ surprisingly, macrophage numbers were higher in HNRNPA2B1-deficient mice. Elevated macrophage numbers were unexpected given that suppression of proliferation classically occurs upon exposure to proinflammatory stimuli such as LPS in conjunction with a switch to glycolysis in order to conserve the cell’s metabolic capacity.^52^^,^^53^ Despite being more abundant, KO macrophages were less active as evidenced by lower levels of the co-stimulatory molecules CD86 and MHCII^54–57^ in response to LPS and the lower macrophage-associated proinflammatory cytokine levels observed in the KO mice. The increase in macrophage and neutrophil population numbers in the KOs could be a direct result of the weakened response to stimulus and failure to reach a terminally polarized state, prompting the cells to proliferate at a faster rate to attempt to compensate for the attenuated response. This weakened inflammatory response in HNRNPA2B1-deficient mice proved detrimental when these mice were infected with *Salmonella*. We chose *Salmonella* as it readily infects macrophages that provide a replicative niche that is abolished through activation of IFNG signaling. Upon pathogen recognition, macrophages polarize to an M1 state that activates T cells and prompts IFNG production; macrophages respond to secreted IFNG through the IFNGR leading to activation of pyroptotic cell death and pathogen clearance.^45^^,^^58^^,^^59^ Following *Salmonella* infection, the HNRNPA2B1-deficient mice succumbed earlier to infection and displayed higher bacterial burden and lower cytokine production indicative of failure to clear the pathogen. HNRNPA2B1-deficient mice fail to induce pyroptosis and instead die through apoptosis, a less inflammatory form of cell death. All these impacts together enable *Salmonella* to replicate more inside the HNRNPA2B1-deficient mice.

Mechanistically we believe that some of these observed phenotypes can be explained by our transcriptomic and splicing analysis. We observed an overall dampening of the IFN responses including downregulation of receptors *Ifngr* and *Ifnar2* and adaptor proteins *Jak1*, *3* and *Stat1*, *3*, as well as transcription factors (*Irf1*, *2*, *7*, *8*, *9*). We report strict nuclear localization of HNRNPA2B1 in macrophages at steady state and following stimulation with a variety of PRR ligands as well as Sendai virus and murine gamma herpesvirus (MHV) (Fig. 5). A recent study by Wang et al. showed that HNRNPA2B1 acts as a DNA receptor and can translocate to the cytosol following HSV-1 infection, influencing IFN signaling through activation of TBK1-IRF3.^17^ It is possible that movement of HNRNPA2B1 is stimulus dependent.

Examination of the splicing landscape of the HNRNPA2B1-deficient mice revealed that several important immune genes show an increase in nonproductive splicing transcripts (NGO transcripts lacking start sites) including the IFNG receptor (*Ifngr1)*, Stat1 and 3, OAS3, and IRF7. While we did not detect noticeable differences in the total protein levels of STAT1, 3, or IRF7, we did capture a decrease in expression of the IFNG receptor at the cell surface of HNRNPA2B1 KO macrophages, as measured by flow cytometry. The observed increase in NGO transcripts represents only a small fraction of total detected isoforms that could contribute to the overall amount of protein expressed. It is technically difficult to capture NGO and other isoforms owing to their potentially rapid degradation, leading to their underrepresentation in our data. Frankiw et al.^60^ demonstrated this phenomenon with the antiviral gene *Oas1g*, where a specific splice site led to transcript decay via NMD. Removing this site increased *Oas1g* expression and enhanced the antiviral response, highlighting the importance of splicing regulation for balanced immune responses, as *Oas1g* overexpression led to increased apoptosis. Analogously, the instability of alternatively spliced isoforms like NGO could lead us to underestimate the targets of HNRNPA2B1.

Given the strong phenotype in the deficient mice in response to *Salmonella* and the importance of IFNG in vivo, we were prompted to further investigate the *Ifngr* effects. Since we confirmed that there was less IFNG receptor present at the cell surface of the KO macrophages, IFNG is no longer capable of coordinating cytokine activation^61–64^ and sensitization of macrophages to potentiate a strong inflammatory response resulting in reduced effector cell (T cells and NK cells) activation and an inability to clear the pathogen. Additionally, the KO macrophages were less responsive to exogenous IFNG stimulation failing to control *Salmonella* replication compared to CTL macrophages. It is also known that IFNG signaling in Salmonella-infected macrophages is essential for activation of inflammasome-mediated pyroptotic cell death,^45^ which releases the pathogen through the lysis of infected cells resulting in the activation of potent inflammatory response machinery. The fact that HNRNPA2B1 macrophages die more readily by apoptosis in lieu of pyroptosis, even in the presence of strong pyroptotic signals, confirms the importance of HNRNPA2B1’s regulation of the IFNGR to promote optimal responses in macrophages.

Clearly there are also additional mechanisms by which HNRNPA2B1 contributes to immune gene expression. Only approximately 10% of genes showed differential splicing patterns in genes that were considered up- or downregulated at the RNA levels by HNRNPA2B1. This suggests that HNRNPA2B1 plays other regulatory roles in controlling these genes outside of splicing. As mentioned earlier many HNRNP proteins play roles at the level of transcription, RNA export, modifications, translation, and splicing, and this is likely also true in the case of HNRNPA2B1.^10^^,^^11^

In conclusion, our work establishes HNRNPA2B1 as a pivotal regulator of innate immunity. We provide novel insights into how a ubiquitously expressed RNA-binding protein can exert cell-type–specific control over immune gene expression and bacterial clearance within macrophages.

## Supplementary Material

vkaf073_Supplementary_Data
