# Supplementary material for: Myeloid-specific HNRNPA2B1 deficiency disrupts macrophage function and in vivo responses
Source: J Immunol. 2025 May 25;214(8):2041–54. doi: 10.1093/jimmun/vkaf073 (PMC12394992; doi:10.1093/jimmun/vkaf073)
Supplement: vkaf073_Supplementary_Data [file vkaf073_supplementary_data.zip › Supplementary figures and legends_final.docx]

**
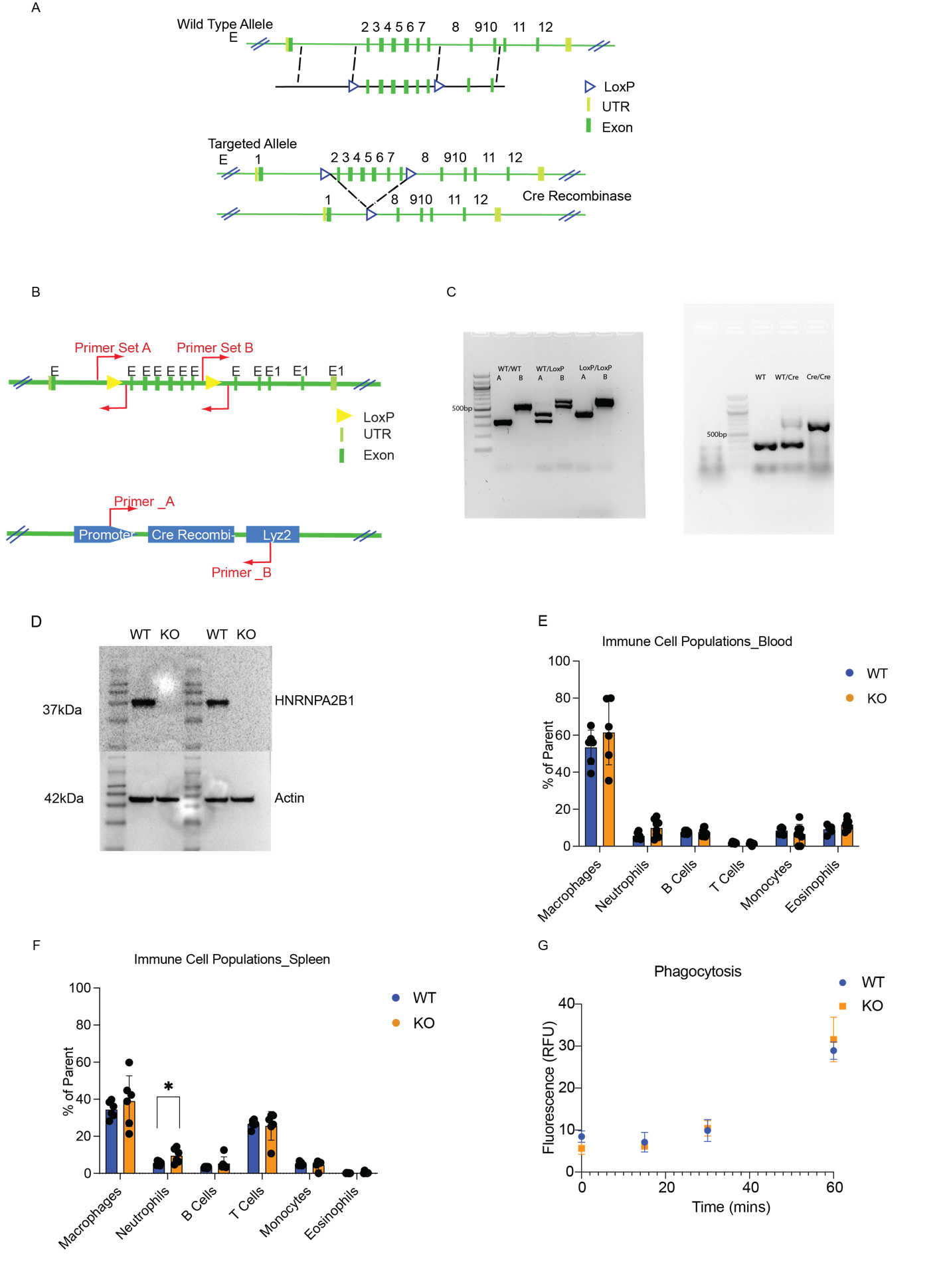
Supplementary figure 1. Generation and characterization of the HNRNPA2B1 conditional knockout mouse. A.** CRISPR was used to add loxP sites on the 5’ and 3’ ends around exons 2 and 7 of the HNRNPA2B1 locus. **B.** Primer design for primer sets A and B which identify successful integration of the loxP sites flanking exons 2 and 7. And primer design to identify successful integration of cre recombinase downstream *Lyz2* promoter. **C.** Genotyping gel showing band sizes for HNRNPA2B1 locus as CTL, heterozygous for loxP and homozygous for loxP (left panel). Genotyping gel showing band sizes for CTL, Cre heterozygous and Cre homozygous locus (right panel). **D.** Western blot analysis of HNRNPA2B1 levels in murine BMDMs. **E.** Profiling of the immune cell repertoire in the blood of HNRNPA2B1 KO mice. **F.** Profiling of the immune cell repertoire in the spleen of HNRNPA2B1 KO mice. G**.** PHrodo green *E. coli* bioparticle assay was used to assess BMDM phagocytic function.


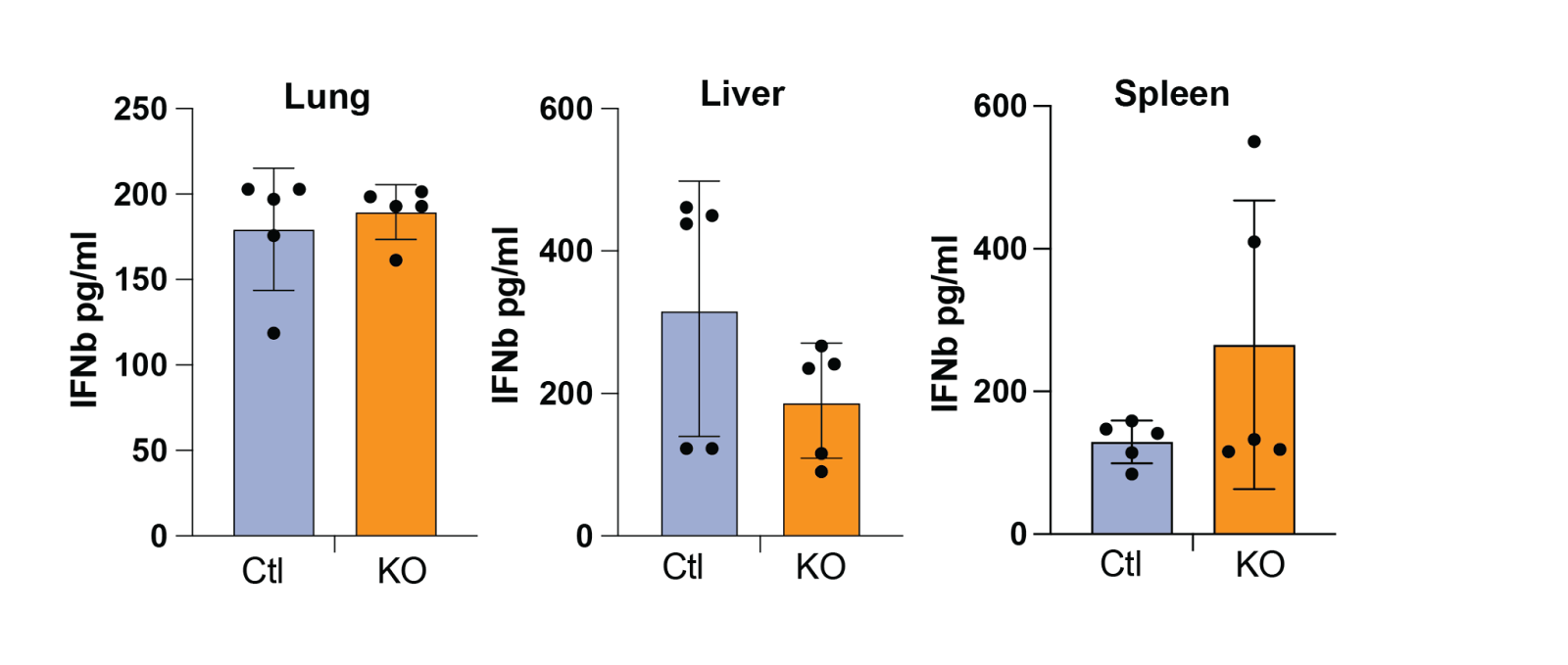


**Supplementary figure 2. IFNb protein levels are not altered in HNRNPA2B1 knockout samples.** Mice were injected with 5mg/kg LPS for 18 h and organs were removed and IFNb levels were measured by Elisa.


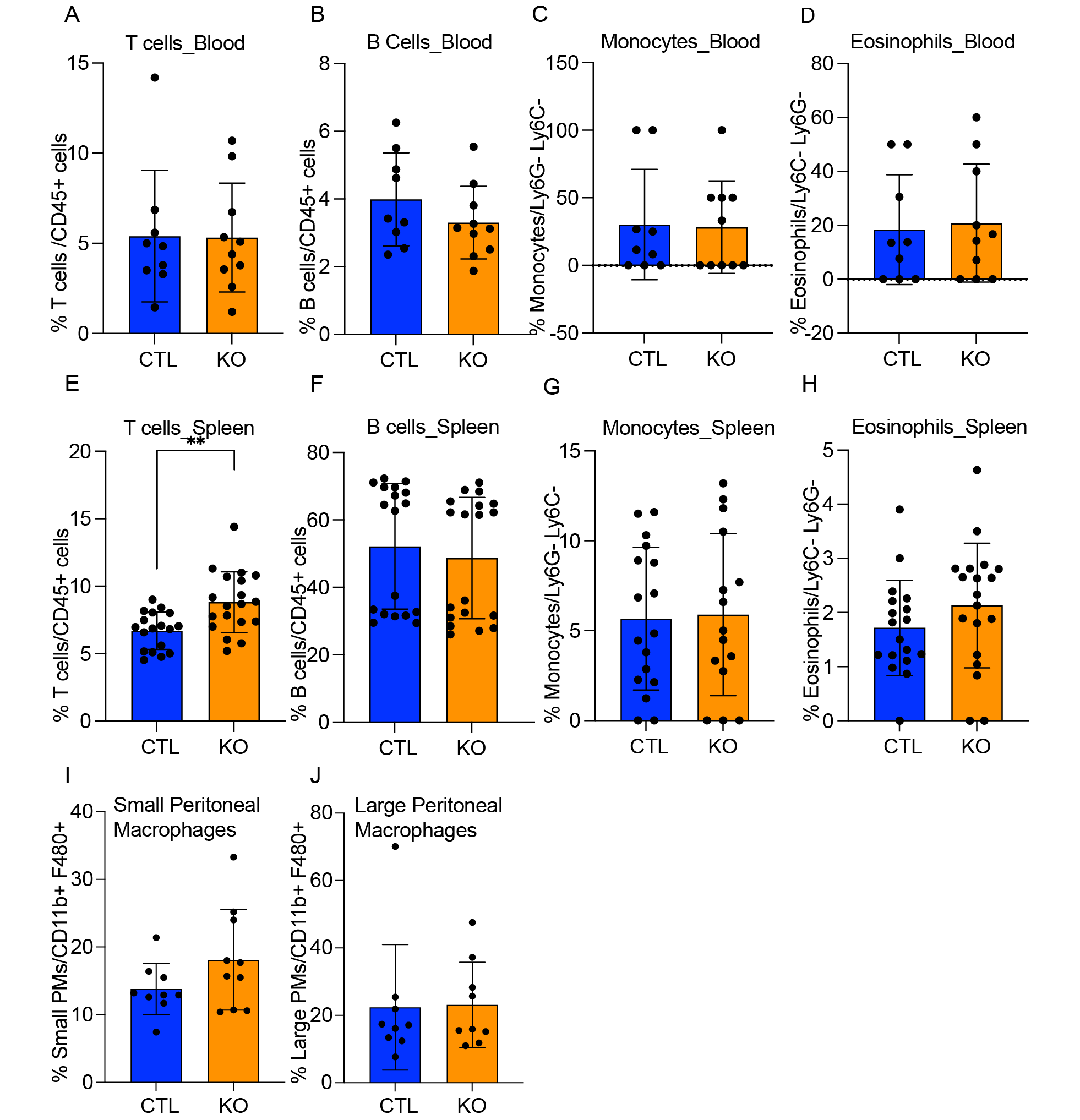


**Supplementary figure 3. Immune cell profiling in blood and spleen of CTL and KO mice. A-D.** Immune cell levels in CTL and KO mice blood analyzed using flow cytometry **A.** T cells**, B.** B cells**, C.** Monocytes**, D.** Eosinophils**. E-H.** Immune cell levels in CTL and KO mice spleen analyzed using flow cytometry **E.** T cells**, F.** B cells**, G.** Monocytes**, H.** Eosinophils**. I.** Levels of small peritoneal macrophages**, J.** Levels of large peritoneal macrophages.


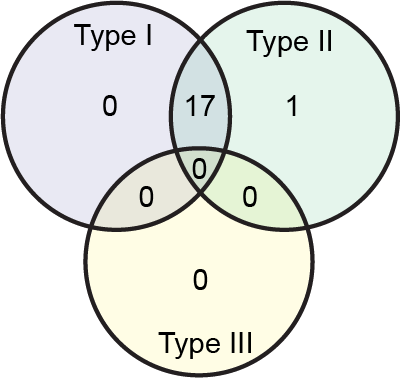


**Supplementary figure 4. Specific interferons are downregulated in HNRNPA2B1 deficient macrophages.** Venn diagram of macrophage interferon subtyping (I,II,III) for downregulated genes in HNRNPA2B1 deficient BMDMs following LPS stimulation.


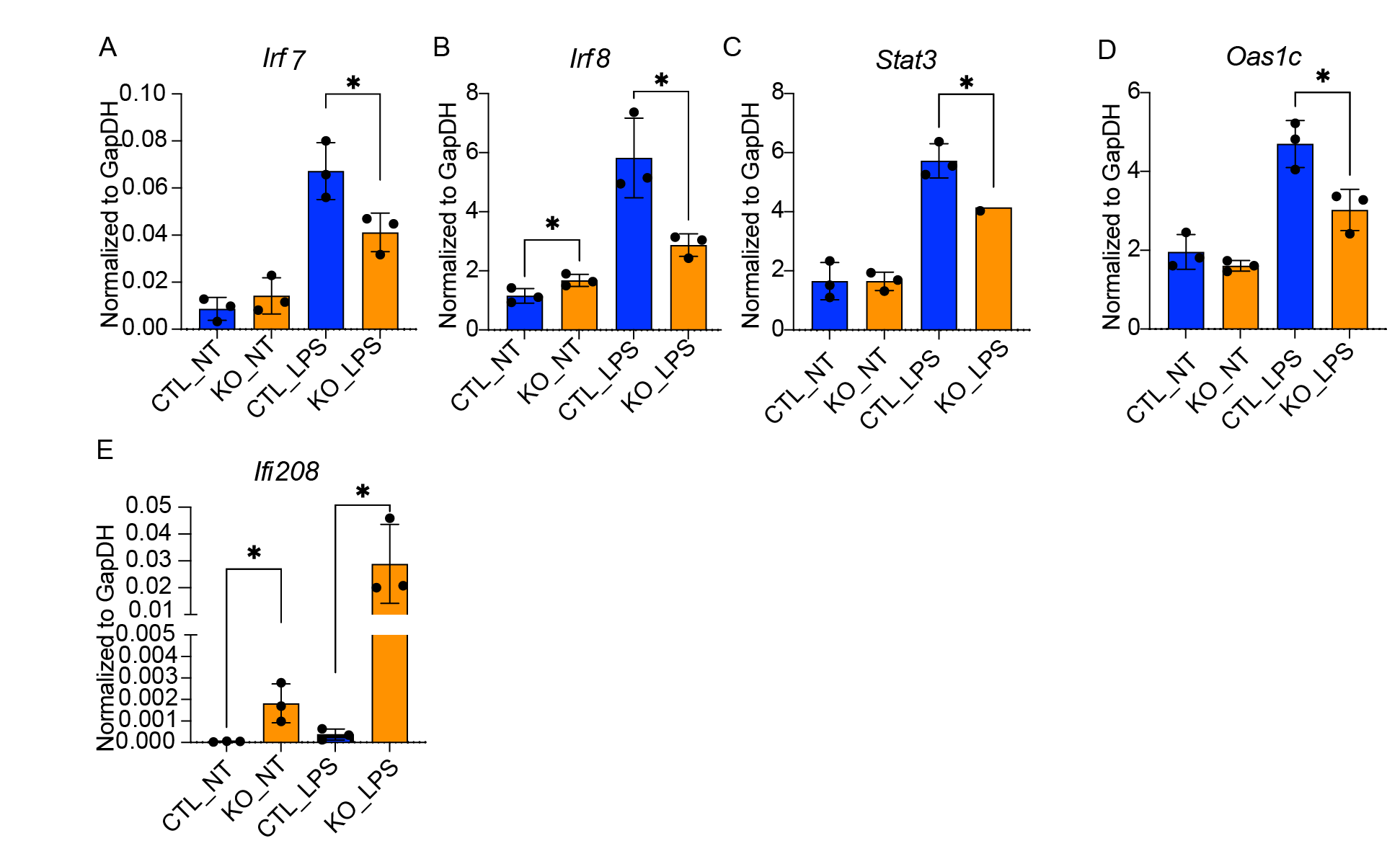


**Supplementary figure 5. HNRNPA2B1 regulates IFN response genes transcriptionally. A-E.** Normalized qRTPCR results for **A.** *Irf7***, B.** *Irf8***, C.** *Stat3***, D.** *Oas1c* and **E.** *Ifi208* Showing changes in CTL and KO BMDMs at baseline and after LPS stimulation.


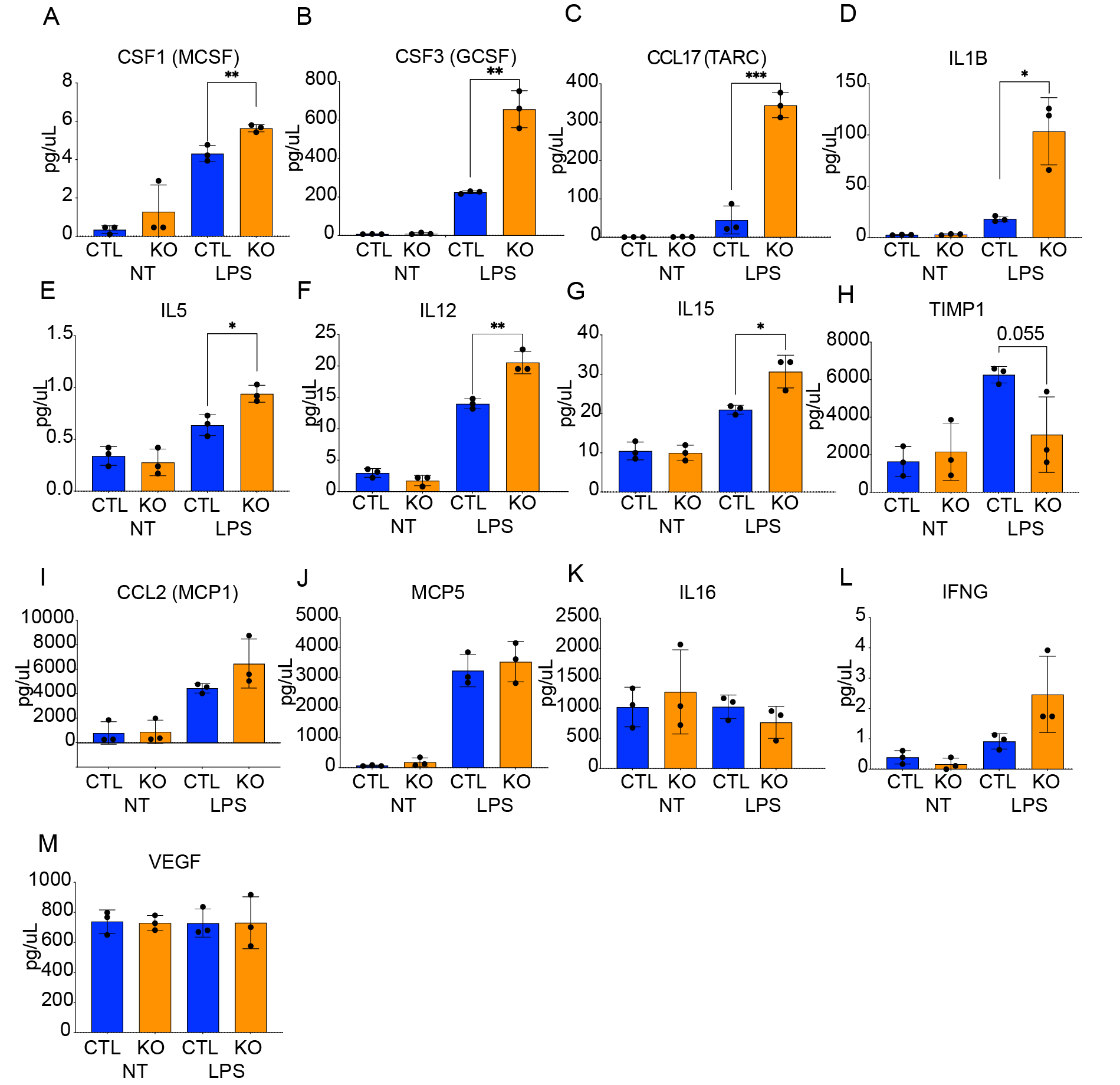


**Supplementary figure 6. HNRNPA2B1 regulates inflammatory cytokine production under stimulus. A-M.** Multiplex ELISA results of inflammatory cytokine changes in CTL and KO BMDM supernatant after treatment with LPS for 18 hrs. **A**. MCSF, **B.** GCSF, **C.** TARC, **D.** IL1B, **E.** IL5, **F.** IL12, **G.** Il15, **H.** TIMP1, **I.** MCP-1, **J.** MCP-5, **K.** Il16, **L.** IFNG, **M.** VEGF. Student’s t-tests were performed using GraphPad Prism. Asterisks indicate statistically significant differences between mouse lines (*P ≤ 0.05, **P ≤ 0.01).


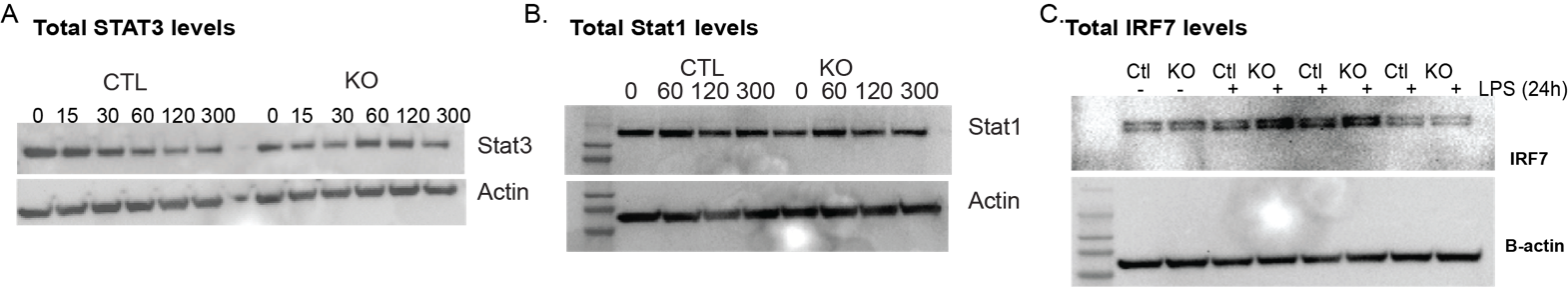


**Supplementary figure 7. HNRNPA2B1 does not impact STAT3, STAT1 or IRF7 total protein levels. A-C.** Western blot analysis of total STAT3, STAT1 and IRF7 levels in HNRNPA2B1 deficient (knockout, KO) or control (CTL) BMDMs following stimulation with LPS (200ng/ml) for the indicated time points.


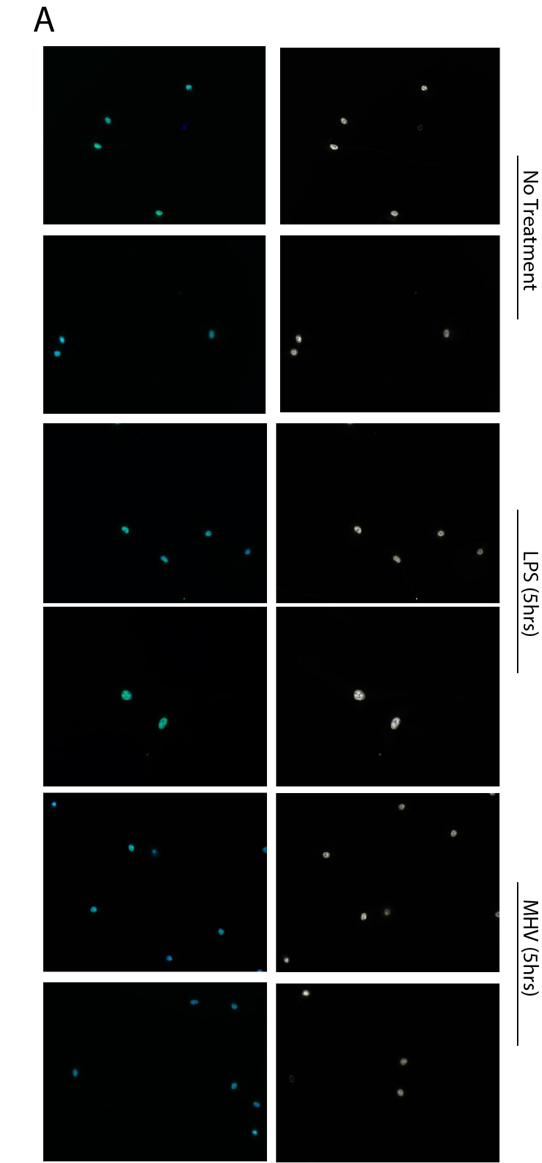


**Supplementary figure 8. HNRNPA2B1 remains in the nucleus at baseline and after exposure to stimulus. A.** Immunofluorescence Images of primary BMDMs. Nuclei stained with DAPI, HNRNPA2B1 stained with an anti-mouse secondary antibody conjugated to Alexa 488. Image processing using Fiji, nuclei boundaries were drawn using masks on DAPI channel and overlaid on to green channel to show that HNRNPA2B1 exists exclusively in the nucleus.


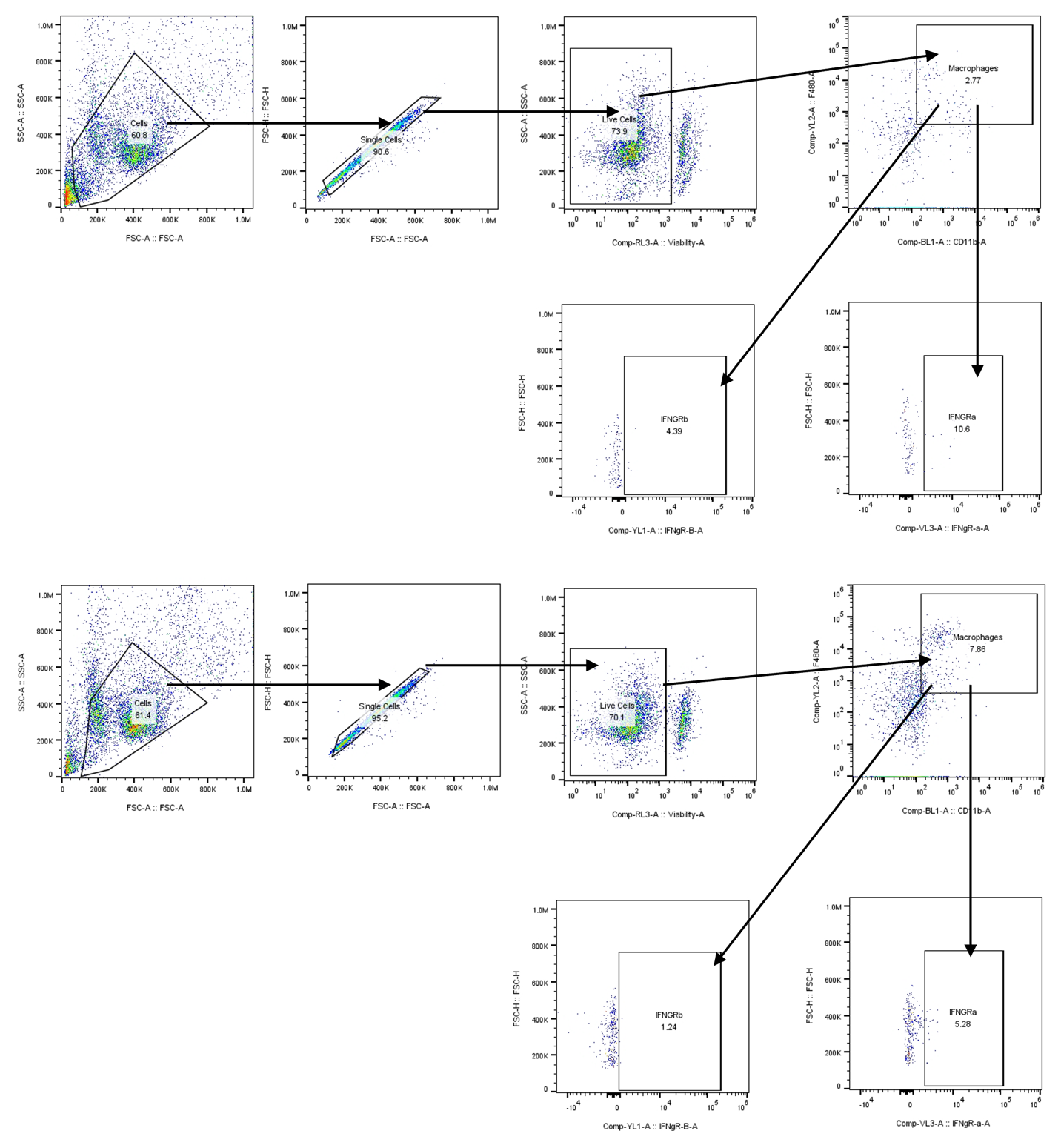


Control Spleen Sample

Knockout Spleen Sample

**Supplementary figure 9. Gating strategy to measure surface expression of IFNGa and b receptors in spleens.** Gates were first set using unstained samples and baseline samples. Mice were injected with LPS (10 mg/kg) and spleens were removed. Here we provide a sample of one control and one knockout spleen gating strategy. First cells were selected on forward and size scatter. Singlets were selected and a live stain was used. Macrophages were selected using CD11b and F480 expression and IFNG a and b receptors were measured.


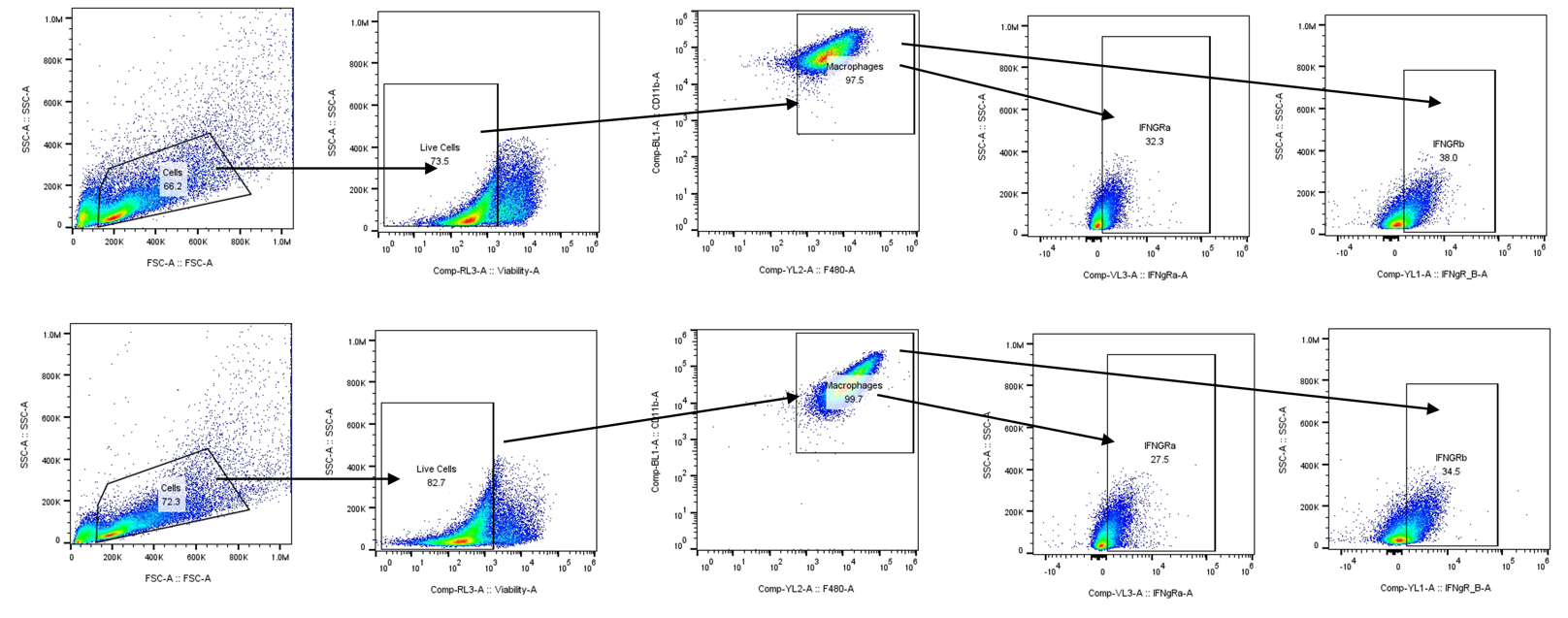


Control BMDM Sample

Knockout BMDM Sample

**Supplementary figure 10. Gating strategy to measure surface expression of IFNGa and b receptors in BMDMs.** Gates were first set using unstained samples and florescence minus one control samples. BMDMs were isolated from control and knockout mice. Here we provide a sample of one control and one knockout BMDM sample. First cells were selected on forward and size scatter. A live/dead stain was used to select on live cells. Macrophages were selected using CD11b and F480 expression and IFNG a and b receptors were measured.
